# Piecewise Disassembly of a Large-Herbivore Community across a Rainfall Gradient: The UHURU Experiment

**DOI:** 10.1371/journal.pone.0055192

**Published:** 2013-02-06

**Authors:** Jacob R. Goheen, Todd M. Palmer, Grace K. Charles, Kristofer M. Helgen, Stephen N. Kinyua, Janet E. Maclean, Benjamin L. Turner, Hillary S. Young, Robert M. Pringle

**Affiliations:** 1 Department of Zoology and Physiology, University of Wyoming, Laramie, Wyoming, United States of America; 2 Department of Botany, University of Wyoming, Laramie, Wyoming, United States of America; 3 Mpala Research Centre, Nanyuki, Kenya; 4 Department of Biology, University of Florida, Gainesville, Florida, United States of America; 5 Division of Mammals, National Museum of Natural History, Washington, District of Columbia, United States of America; 6 Department of Wildlife Management, Moi University, Eldoret, Kenya; 7 Department of Zoology, University of British Columbia, Vancouver, British Columbia, Canada; 8 Smithsonian Tropical Research Institute, Balboa, Ancon, Panama; 9 Department of Organismic and Evolutionary Biology, Harvard University, Cambridge, Massachusetts, United States of America; 10 Department of Ecology and Evolutionary Biology, Princeton University, Princeton, New Jersey, United States of America; University of Fribourg, Switzerland

## Abstract

Large mammalian herbivores (LMH) strongly influence plant communities, and these effects can propagate indirectly throughout food webs. Most existing large-scale manipulations of LMH presence/absence consist of a single exclusion treatment, and few are replicated across environmental gradients. Thus, important questions remain about the functional roles of different LMH, and how these roles depend on abiotic context. In September 2008, we constructed a series of 1-ha herbivore-exclusion plots across a 20-km rainfall gradient in central Kenya. Dubbed "UHURU" (Ungulate Herbivory Under Rainfall Uncertainty), this experiment aims to illuminate the ecological effects of three size classes of LMH, and how rainfall regimes shape the direction and magnitude of these effects. UHURU consists of four treatments: total-exclusion (all ungulate herbivores), mesoherbivore-exclusion (LMH >120-cm tall), megaherbivore-exclusion (elephants and giraffes), and unfenced open plots. Each treatment is replicated three times at three locations (“sites”) along the rainfall gradient: low (440 mm/year), intermediate (580 mm/year), and high (640 mm/year). There was limited variation across sites in soil attributes and LMH activity levels. Understory-plant cover was greater in plots without mesoherbivores, but did not respond strongly to the exclusion of megaherbivores, or to the additional exclusion of dik-dik and warthog. Eleven of the thirteen understory plant species that responded significantly to exclusion treatment were more common in exclusion plots than open ones. Significant interactions between site and treatment on plant communities, although uncommon, suggested that differences between treatments may be greater at sites with lower rainfall. Browsers reduced densities of several common overstory species, along with growth rates of the three dominant *Acacia* species. Small-mammal densities were 2–3 times greater in total-exclusion than in open plots at all sites. Although we expect patterns to become clearer with time, results from 2008–2012 show that the effects of excluding successively smaller-bodied subsets of the LMH community are generally non-additive for a given response variable, and inconsistent across response variables, indicating that the different LMH size classes are not functionally redundant. Several response variables showed significant treatment-by-site interactions, suggesting that the nature of plant-herbivore interactions can vary across restricted spatial scales.

## Introduction

Human activities have driven thousands of species extinct and extirpated tens of thousands of populations [1]–[3]. The direct and indirect ramifications of these extirpations for other species can be profound, and the implications for community structure and ecosystem functioning are difficult to anticipate. Our inability to predict the ecological implications of species loss reflects a lack of basic understanding about the functional roles of even large, well-studied species.

This shortfall is compounded by the fact that the outcomes of species interactions differ, in magnitude and even direction, as a function of environmental context–which itself varies in space and time [4]–[11]. Thus, experimental findings from different systems often fail to align, and it remains difficult for researchers to extrapolate beyond the restricted spatial and temporal scales at which most experiments are conducted [12], [13]. Resolving the challenges posed by contingency has therefore been identified as a central goal of ecology [4], [10]. Doing so will require a variety of strategies, including large-scale observational approaches and meta-analytic syntheses. However, it is also necessary to expand the geographic and temporal scope of field experiments by simultaneously imposing identical manipulations in locations that differ along one or more key axes of environmental variation.

Among these key axes are precipitation regimes, which are rapidly shifting with global climate change. Alteration of precipitation regimes, the intensity of droughts, and the number of extreme rainfall events are anticipated to have a profound impact on terrestrial ecosystems [14], perhaps particularly so for the ∼40% of the terrestrial land surface [15] that is classified as arid or semi-arid. For example, the quantity and temporal distribution of rainfall events determine patterns of primary productivity in grassland ecosystems [16], and variation in rainfall can cause changes in species abundances, thus altering the strength of density dependence and other regulatory processes [17], [18]. Likewise, drought may interact with temperature increases to depress plant and animal populations [19].

African savannas have long fascinated scientists and the public alike, and both rainfall and species interactions are essential in maintaining the structure and function of these ecosystems. Most conspicuously, the co-dominance of trees and grasses that defines savannas is often unstable, existing in a non-equilibrial state that is determined by the interplay of rainfall, fire, and large mammalian herbivores (LMH, ≥5-kg) [20]–[23]. Due to their large body sizes, long generation times, valuable meat and body parts, and capacity to conflict with rural livelihoods, LMH are disproportionately prone to anthropogenic population declines and extirpation [24], [25]. Such declines typically proceed in a size-biased fashion, with bigger species disappearing first [24], [26]. It is therefore important to understand how savanna structure and function respond to the loss of successively smaller size classes of LMH.

Prior studies have shown that changes in LMH abundance (both in Africa and elsewhere) can strongly influence a wide range of other taxa, community properties, and ecosystem processes. Examples include direct and indirect effects on the productivity, density, diversity, recruitment, reproduction, and individual traits of plants (reviewed in [27]–[34]), as well as indirect effects on populations and assemblages of insects, small mammals, and other consumers (e.g., [35]–[44]). However, most experimental studies have utilized an “all-or-none” approach of excluding entire LMH guilds (but see [45]–[47]), leaving uncertainty about which species are responsible for which effects. Moreover, the expense and sampling effort involved in large-scale LMH manipulations is such that very few studies have simultaneously applied identical treatments in multiple locations along environmental gradients (but see [48]–[50]). Meta-analyses and meta-experiments conducted at continental or intercontinental scales have investigated the generality of some of the aforementioned effects of LMH; intriguingly, results suggest that the direction and magnitude of effects are often contingent on local resource availability [51]–[53]. Yet such broad synthetic approaches have limitations, including difficulties in mechanistic inference, biases arising from both study-selection criteria and differences in the scale/methods of individual studies, and a tendency to gloss over potentially important local contingency by focusing on overall trends [10], [54], [55].

Hence, there is an important role for large-scale field experiments that selectively disassemble LMH communities across environmental gradients that are not confounded by dramatic differences in other biotic and abiotic attributes. Such experiments will enable us to identify the respective impacts on plants and animals of different subsets of LMH communities; to evaluate whether smaller herbivores can functionally compensate for the loss of larger ones; to assess how resource availability mediates these impacts; and to help develop a mechanistic understanding of context dependence. The need for such studies was articulated in a recent synthesis of consumer vs. resource control of producer biomass [56], which urged “implementation, particularly in terrestrial systems, of standardized, replicated field experiments across a spatial network of sites that can serve as standardized tests of trends revealed through meta-analysis.”

In September 2008, we initiated a replicated large-herbivore exclusion experiment, dubbed “UHURU” (Ungulate Herbivory Under Rainfall Uncertainty). The overarching objectives of UHURU are: to selectively exclude successively smaller-bodied subsets of the LMH guild from 1-ha plots in a way that mimics size-biased extinction and isolates the impacts of different groups of LMH species; to replicate these plots at a spatial scale large enough to encompass a biologically meaningful gradient in rainfall, yet small enough that all sites share similar edaphic characteristics and species drawn from the same regional pool; and to test predictions about the independent and interactive effects of LMH exclusion and rainfall variability on a broad range of response variables.

Here we provide a thorough description of the experimental design and initial conditions (thus laying the groundwork for future contributions) and report results from the first 3.5 years of the experiment (thus broadly characterizing the short-term responses of savanna communities to altered herbivory regimes). Our work was guided by the following hypotheses: (1) LMH suppress densities of most plants and small mammals, but may facilitate some plant species by reducing competitive dominance; (2) mesoherbivores exert particularly strong effects because, collectively, they are both abundant and functionally diverse in terms of foraging mode (comprising grazers, browsers, and mixed feeders); (3) suppressive effects of LMH on plant densities are strongest in lower-rainfall sites because plants there are less able to tolerate herbivory, whereas facilitative effects are strongest in high-rainfall sites because the potential for competitive exclusion is greater.

## Methods

### Study Site and Experimental Design

**Figure 1:**
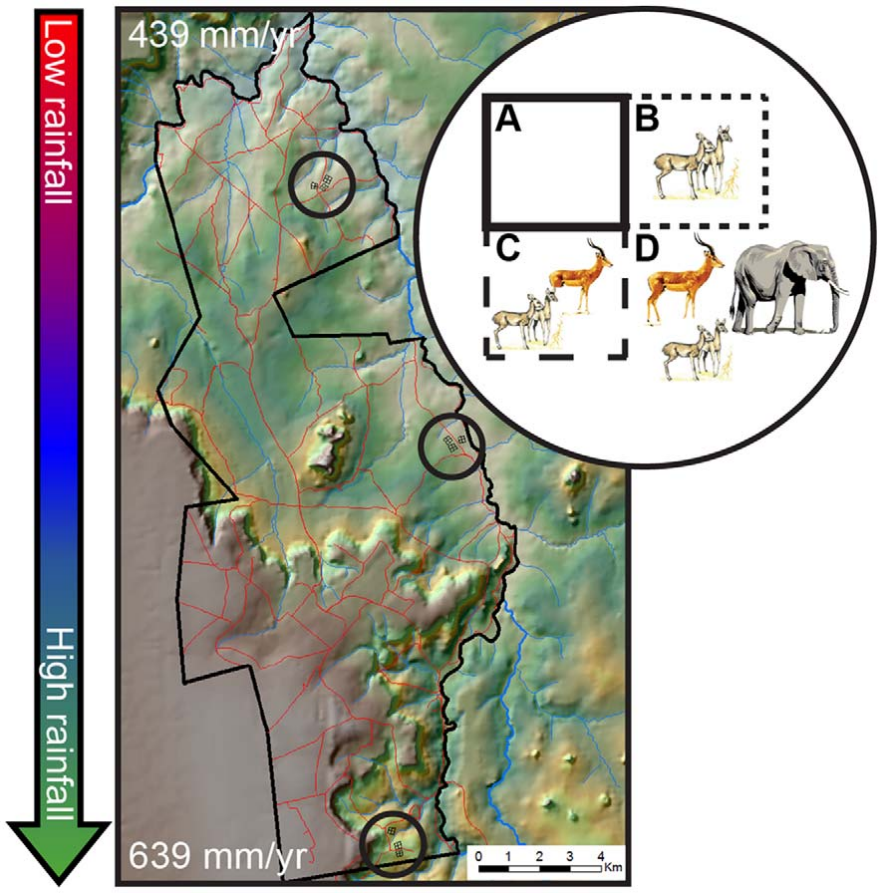
Terrain map of Mpala Research Centre showing north-south rainfall gradient and schematic of the experimental design. Three blocks, each containing one 100×100-m replicate of each treatment, are situated at each circled location on the map; 20 km separates the northern (low-rainfall) and southern (high-rainfall) sites. Red lines indicate dirt roads. (A) Total-exclusion plots exclude all large herbivores; (B) mesoherbivore-exclusion plots exclude all herbivores larger than warthogs; (C) megaherbivore-exclusion plots exclude elephants and giraffes only; (D) open plots are accessible to all species.

Our research is conducted at the Mpala Research Centre, part of a private conservancy in Laikipia County, central Kenya (0°17′N, 37°52′ E, 1600-m elevation). All work was conducted with permission from the Kenyan government (permit NCST/5/002/R/656), the Director of Mpala Research Centre, and IACUC protocol SKMBT-60112030515200 (University of Wyoming). Mpala is located northwest of Mount Kenya and falls in its rain shadow, leading to pronounced climatic variability at relatively small spatial scales: from 2009–2011, mean annual rainfall increased >45% over 20 km from north to south (Fig. 1). The soils occurring across this gradient, characteristic of the region, are infertile red sandy loams derived from metamorphic basement rock [57]. The soils are classed as Alfisols (Typic Haplustults) according to US Soil Taxonomy [58] and support a discontinuous understory of grasses and forbs [59]. The overstory is dominated by three species of *Acacia* (*A. etbaica*, *A. brevispica*, *A. mellifera*). Fires are infrequent, limited by both the discontinuous understory and active suppression of anthropogenic fires by land managers since the mid-1900s [60], [61]. Twenty-two species of native large herbivores occur at Mpala, along with a diverse carnivore community (Table S1).

The UHURU experiment consists of four herbivory treatments that were randomly assigned to contiguous 1-ha (100×100 m) plots (Fig. 1) [62]. “Total” excludes all herbivores larger than ∼5-kg mass and ∼50-cm tall (but is accessible to hares and other small mammals), using 2.4-m high fences consisting of 14 strands of electrified wire with a 1-m high chain-link barrier (10-cm mesh) at ground level (Fig. S1a). “Meso” consists of 11 wires starting ∼30-cm above the ground, allowing access by LMH <120 cm tall (dik-dik *Madoqua cavendishi* and warthog *Phacochoerus africanus*), but excluding all larger LMH species (Fig. S1b). “Mega” consists of two wires 2-m above ground level, excluding only megaherbivores (elephants *Loxodonta africana* and giraffes *Giraffa camelopardalis*; Fig. S1c). “Open” plots are unfenced; a series of 1-m tall wooden posts at 10-m intervals demarcates plot boundaries (Fig. S1d). On all fences, a series of 1-m long wires at 2-m height extend horizontally outward from the plots to deter elephants and giraffes that approach the barriers (Fig. S1a-c). In January 2009, vertical connecting wires were added to total- and mesoherbivore-exclusion fences to prevent impala and zebra from passing between the horizontal wires.

Three blocks (each containing one randomly assigned replicate of each treatment) are located at each of three sites along the rainfall gradient (“Low”, “Intermediate”, and “High”). The experiment thus comprises a total of 36 1-ha plots: 4 plots/block × 3 blocks/site × 3 sites. Collectively, these treatments allow us to evaluate the effects of LMH species spanning three orders of magnitude in body mass, from dik-dik (4–6 kg) to elephant (3000–7000 kg). In each plot, we established a central 60×60 m grid of 49 rebar stakes at 10-m intervals; this grid serves as the basis for much of our sampling.

At Mpala, there is a single dominant LMH species (in terms of biomass density) within each LMH size class distinguished by the experiment [63]: dik-dik in the smallest group, impala (*Aepyceros melampus*) in the intermediate group, and elephant in the largest group. The estimated total energetic requirements of these three species–derived from published Mpala-wide density estimates [63] coupled with the metabolic-rate equations of Nagy et al. [64]–is roughly equivalent (Fig. S2).

Each treatment in UHURU can be compared with the unfenced Open plots to gauge the effects of all LMH up to a certain size, or can be assessed relative to other treatments to isolate the effects of a given LMH size class. For example, the Mega vs. Open comparison isolates the largest size class (elephants and giraffes); likewise, the only difference between Total and Meso plots is the presence of the smallest size class (comprising dik-dik and warthogs), and comparisons between these plots should largely reflect the impacts of dik-dik (which are far more common than warthog). The mesoherbivore category is the most heterogeneous, comprising eight species known to occur in the plots. In order of decreasing abundance, these are: impala, plains zebra (*Equus quagga*), eland (*Taurotragus oryx*), Grevy’s zebra (*E. grevyi*), waterbuck (*Kobus defassa*), buffalo (*Syncerus caffer*), oryx (*Oryx beisa*), and gerenuk (*Litocranius walleri*). Of these, only the first four are common, and impala are far more common than the rest [63]. Cattle and camels are ranched at low densities on Mpala [46], but herders are asked not to allow livestock within the plots, allowing us to focus on the effects of wild herbivores.

### Monitoring LMH Activity

Every three months, we conducted dung surveys to assess the effectiveness of the experimental treatments and to provide an index of LMH activity levels. In each plot, we established three 60×5 m belt transects, parallel and spaced 30-m apart within the central 60×60 m grid. During surveys, two observers walked these transects, counting all discrete dung piles and identifying the species of origin [65]. Dung was crushed after identification to prevent its being recounted in subsequent surveys. Because we were unable to distinguish between the dung of plains and Grevy’s zebras, we lumped these two species in analyses. Because inferences about LMH activity levels could be biased by differential dung-decomposition rates in wetter vs. drier locations, we assessed decomposition rates of dik-dik, impala, and elephant dung at each site within UHURU. In November 2011, we placed 10 fresh dung piles at 10-m intervals along a 100-m transect near the experimental plots at each site. Dung piles were weighed prior to placement, and observers noted the amount of understory cover and sun exposure (both classified as none, partial, or full) where placement occurred. Thirty days later, we collected and weighed what remained of each dung pile.

To help produce a more comprehensive list of the LMH species present in the plots, we supplemented these dung-count data with periodic bouts of camera-trap sampling. Two infrared camera traps (Reconyx RM45) were deployed in opposite corners of each plot for two weeks at a time at two-month intervals during 2010–2011 (camera settings: “medium/high” sensitivity, 3 pictures per trigger, rapidfire interval, no delay period).

### Abiotic Environment

Rainfall has been continuously monitored at each of the three sites along the rainfall gradient. Starting in October 2008, rainfall was measured using cylindrical drip gauges (All Weather Rain Gauge, Productive Alternatives, Fergus Falls, MN). In June 2010, we installed a single automated tipping-bucket rain gauge (RainLogger, Rainwise Inc., Bar Harbor, ME) within one of the Total-exclusion plots at each site. We installed a second tipping-bucket gauge at each site in July 2011 and a third in April 2012. Six of the tipping-bucket gauges were calibrated in August 2011, with mean error for each gauge ranging from −0.4% to +5.3%.

Soils were classified by manually excavating a profile pit to bedrock near the exclusion plots at each site. The profiles were described according to USDA Soil Taxonomy [58]. Bulk density was assessed by the excavation method [66], removing ∼1 L of soil and measuring the excavated volume of the plastic-lined hole with water. Samples were taken by genetic horizon, air-dried, and returned to the Smithsonian Tropical Research Institute in the Republic of Panama for physical and chemical analyses. Soil pH was determined using a glass electrode in both deionized water and 0.01 M CaCl_2_ in a 1∶2 soil-to-solution ratio, as well as in 0.1 M BaCl_2_ extracts at a 1∶30 soil-to-solution ratio. Particle size distribution was determined by the pipette method following pretreatment to remove soluble salts, organic matter, and iron oxides [67]. Total carbon and nitrogen were determined by automated combustion and gas chromatography with thermal conductivity detection using a Thermo Flash 1112 analyzer (CE Elantech, Lakewood, NJ, USA). Total phosphorus was determined by ignition (550°C, 1 h) and extraction in 1 M H_2_SO_4_ (16 h, 1∶50 soil to solution ratio), with phosphate detection by automated neutralization and molybdate colorimetry on a Lachat Quikchem 8500 (Hach Ltd, Loveland, CO, USA). Exchangeable cations were determined by extraction in 0.1 M BaCl_2_ (2 h, 1∶30 soil to solution ratio), with detection by inductively-coupled plasma optical-emission spectrometry on an Optima 7300 DV (Perkin-Elmer Ltd, Shelton, CT, USA) [68]. Total exchangeable bases (TEB) were calculated as the sum of Ca, K, Mg, and Na; effective cation exchange capacity (ECEC) was calculated as the sum of Al, Ca, Fe, K, Mg, Mn, and Na; base saturation was calculated as (TEB ÷ ECEC) × 100.

Various properties of surface soils were measured within the exclosures in each year from 2009–2011. In February 2009, we collected 20-cm deep soil cores at 12 evenly spaced locations around the periphery of the central 60×60 m grid in each plot. All samples from each plot were thoroughly mixed, subsampled, and sent to the World Agroforestry Centre (ICRAF) in Nairobi for analysis (pH, exchangeable Ca, Mg, K, and P, and total percent C and N). In June 2010, we collected 20-cm deep soil cores from the four corners of the central 60×60 m grid in Open and Total plots only. Each of these four samples was individually sealed in a Whirl-Pak bag (Nasco, Fort Atkinson, WI), frozen, and delivered within 96 h to Crop Nutritional Services (Nairobi, Kenya) for analysis of NO_3_, NH_4_, and percent sand, clay, and silt. In January 2012, we again collected 20-cm cores from the four corners of the central 60×60 m grid in open and total-exclusion plots. Samples were dried (65°C for 72 hours), homogenized and sieved through 2-mm mesh, and sent to Brookside Laboratories (New Knoxville, OH) for analysis of pH, organic matter (derived from loss on ignition), percent sand, silt, and clay, and extractable Al, B, Ca, Cu, Fe, K, Mg, Mn, Na, P, S, and Zn. Total percent C and N were also analyzed from the same samples at Stanford University’s Environmental Measurements (EM-I) facility.

Finally, in September-October 2012, we measured soil-infiltration capacity in each plot, following standardized methods of the Land Degradation Surveillance Framework [69]. Briefly, a single 20-cm diameter infiltration ring was hammered into bare soil at the center of each plot. We pre-wetted the soil and then repeatedly filled the ring with water to a level of ∼160 mm over 130 min (at 5-min intervals for the first 30 min, 10-min intervals for the next 60 min, and 20-min intervals for the final 40 min, for a total of 14 successive fillings), recording the beginning and end water level for each time interval. Infiltration rates (mm/min) were recorded for each interval, and mean infiltration rates were calculated for each plot using (a) data from all 14 fillings and (b) data from only the final 5 fillings.

### Proxies for Primary Productivity

Due to spatial heterogeneity in the understory vegetation at our study sites [59], it is difficult to estimate primary production using standard grassland approaches such as the moveable-cage method [70]. We therefore report two proxies for primary productivity. First, we delineated two 8×8 m areas (comprising 64 1-m^2^ cells) within total-exclusion plots at each of the three sites along the rainfall gradient; we selected areas haphazardly, subject to their having continuous understory vegetation and no trees. In January 2012, corresponding to peak biomass following a high-rainfall year, we collected, dried, and weighed all standing vegetation biomass and litter from each grid cell. We calculated the average biomass of the 64 cells in each 8×8 m, yielding two measurements at each site.

Second, we calculated the Normalized Difference Vegetation Index (NDVI) for each plot using a Quickbird satellite image collected in November 2009 (following the short rains); NDVI was calculated for each pixel, and we recorded the maximum, minimum, and mean NDVI values of all pixels within each plot.

### Understory Plant Community

Grasses and forbs were surveyed twice annually in all plots in February/March (dry season) and October (short rains). A 1-m^2^ quadrat was placed immediately to the north of each stake in the central 60×60 m grid, and a 0.25-m^2^ quadrat was placed within it; species presence/absence was recorded within both quadrats. We then centered a 10-pin point frame within the smaller quadrat and recorded the total number of vegetation pin hits for each species, as well as the number of bare-ground hits. Prior work in both this system [71] and others (e.g., [72]) shows that number of pin hits is strongly correlated with understory biomass. Individuals were identified to species (or to genus+morphospecies) with the aid of field guides and published species lists [73]–[75]; these identities are provisional pending ongoing taxonomic work and DNA barcoding. We calculated observed species richness, asymptotic species richness (Chao2 estimator), and Shannon diversity and evenness of understory plants for each plot in each survey.

### Overstory Plant Community

Once per year, we censused all shrubs, trees, and tall succulents within the central 60×60 m grid in each plot. This 3600-m^2^ area is subdivided into 10×10-m cells, which were exhaustively searched by 2–4 observers. Individuals were identified to species using field guides [76], [77] and binned in one of five height classes (≤1 m, 1–2 m, 2–3 m, 3–4 m, ≥4 m). Here we present data from the 2012 survey only, since we did not expect the overstory community to respond immediately to herbivore exclusion.

To assess woody-plant growth rates and other individual-level parameters, we tagged 10 individuals in each plot (or all individuals if <10 occurred in a plot) of five common woody species in January 2009: the three dominant acacias (*A. etbaica*, *A. mellifera*, and *A. brevispica*), *Croton dichogamus* (Euphorbiaceae), and *Balanites aegyptiaca* (Zygophyllaceae). We also tagged all individuals ≥1-m tall of a sixth species, *A. drepanolobium*, which is dominant on nearby black-cotton soils (Vertisols), but rare on sandier soils and restricted to our southern (high-rainfall) sites. Tagged individuals were resurveyed in February of each year. We recorded the following data: height (in cm), canopy area (in cm^2^, estimated as an ellipse based on the length of the longest axis and its perpendicular), and basal circumference (in cm, 15 cm from ground level). We also recorded the number of stems at ground level and any occurrence of elephant damage, and we visually estimated the number of fruits, flowers, and floral buds. Here, we report only total vertical and lateral (canopy) growth over the three-year interval 2009–2012.

### Small-mammal Community

Since May 2009, we have trapped small mammals at two-month intervals in all total-exclusion and open plots (only). In each trapping session, a folding Sherman live-trap was set for four consecutive nights at each of the 49 stakes in the central 60×60 m grid of each plot. Traps were baited with peanut butter and oats, set in the evening, and checked in the morning.

Initial species identifications based on field characters were revised following examination of cranial morphology and DNA barcodes of small mammals collected outside of UHURU as part of a different study (CO1-5P locus; sequencing done at the University of Guelph). Based on field measurements and DNA barcodes, we retroactively corrected the initial classifications of all live-trapped taxa (except for the 2–4 *Mus* spp. and several *Crocidura* spp., which we cannot reliably distinguish in the field; these species are therefore recorded and analyzed only at the genus level). All of the misidentifications involved the classification of *Taterillus harringtoni* as *Gerbilliscus robustus*. We now distinguish these two species based on the following characteristics: (1) mature *G. robustus* exceed 60 g total mass; (2) all *G. robustus* have hindfoot lengths >34 mm; and (3) *G. robustus* lacks a tufted tail. Each live-trapped individual was marked for identification with a Monel fingerling eartag in each ear, except for *Acomys*, *Crocidura*, and *Mus* spp., which are too delicate; these species were instead marked with red marker for individual identification within sampling bouts. Weight, sex, age, and reproductive condition were recorded at the time of capture. Here, we report small-mammal densities as the minimum number known alive (MNKA) of the whole community [18], scaled to an area of 1 ha.

### Statistical Analysis

Descriptive statistics are presented as means ±1 SEM. Unless otherwise specified, we analyzed experimental results using mixed-model analyses of variance (mmANOVA) with site (*n* = 3), treatment (*n = *4 or 2, because some responses were measured only in Open and Total plots), and the treatment*site interaction as fixed effects, and with block (*n = *9) as a random effect. We adopted a conservative statistical approach: in comparisons involving plot-level data, plot-wide means were the units of analysis. Moreover, in most cases where measurements were repeated in multiple years or seasons, we averaged across surveys to produce a synthetic view of the first several years of the experiment (for understory-plant analyses, we also conducted separate analyses for each of the seven surveys conducted from 2008–2011). Most analyses, therefore, had a total of 18 or 36 data points, each corresponding to the average value (over however many sampling locations and intervals) of one plot. Non-normal data were transformed as indicated in the text; this included all dung-count and most understory pin-hit data (which were square-root transformed), as well as dung-decomposition data (for which percent change in weight was arcsine square-root transformed). Although data for some understory plant species remained significantly non-normal (Sharpiro-Wilk *W* test) even after transformation, we nonetheless proceeded with parametric analysis because ANOVA with balanced designs is usually robust to moderate deviations from normality [78], [79]. When a fixed effect with more than two levels was statistically significant (*P*≤0.05), we examined pairwise differences using Tukey’s Honestly Significant Difference (HSD) post-hoc tests. These analyses were conducted in JMP 9.0.2 (SAS, Cary, NC). We did not adjust alpha for the multiple comparisons of soil attributes and understory plants, because we believed that standard corrections increased the probability of Type II error to unacceptable levels. Instead, we present the results of our otherwise conservative analyses with the aim of identifying biologically meaningful trends for further investigation, and we interpret marginally significant results with due caution.

We used Kruskal’s non-metric multidimensional scaling (MDS) to analyze the compositional similarity of surface soils and plant communities. These analyses were conducted using the isoMDS function in the MASS package in R. For surface-soil MDS analyses, we used data on 18 physical and chemical attributes from samples collected in 2012, along with NO_3_ and NH_4_ data from 2010 (open and total-exclusion plots only: see Table S2). For the understory-plant community, we used the total number of pin hits of each plant species in each plot, along with the number of bare-ground pin hits, averaged over the seven surveys from 2008–2012. For the overstory plant community, we used the density of each species in each plot in the 2012 census. We quantitatively tested the compositional similarity of soils and plant communities in R using the adonis function of the vegan package, which conducts permutational MANOVA (perMANOVA) using distance matrices; we specified models with site, treatment, and their interaction as factors, and with 100,000 permutations per test. Rank-abundance curves for under- and overstory plant communities were constructed in R using the rankabundance function in the BiodiversityR package.

For analyses of LMH dung counts, we were concerned with (a) confirming the effectiveness of the experimental barriers (i.e., ensuring that species’ dung was not present in plots from which those species are supposed to be absent, and conversely that barriers do not have unintended negative effects on the activity of non-target species) and (b) looking for variation in activity levels of different species across the three sites along the rainfall gradient. We first summed dung counts within each plot for each survey and averaged across all surveys to obtain a mean dung density for each species in each plot. We then assessed exclosure effectiveness as the percent reduction of dung deposition. To address variation in activity levels across sites, we used mmANOVA (as specified above) for each species, omitting plots from which that species was supposed to be excluded. Any significant effects of treatment in these models (not applicable for megaherbivores, which are present only in open plots) indicates unintended effects of the experimental barriers (i.e., altered activity levels of a given species in treatments not designed to manipulate that species); significant effects of site in these models reflect variation in activity levels across the rainfall gradient.

## Results

### Large-mammal Community

**Figure 2:**
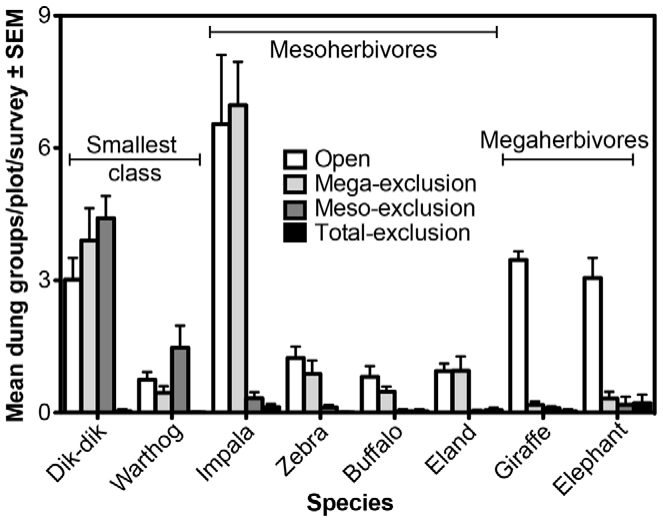
Patterns of dung deposition by the eight most common LMH species, arranged in order of increasing body mass. Data are the mean number of dung groups per plot across eight surveys from April 2009 to November 2011.

As of March 2012, 13 native LMH species, along with two domestic species and 14 carnivores, had been recorded in the plots (Table S1). Dung surveys showed that the experimental treatments were highly effective. No species’ dung was found in appreciable quantity in plots from which that species was excluded (Fig. 2); for the eight most common LMH, exclosure effectiveness ranged from 92% (for elephants) to 99% (for warthog and dik-dik; mean effectiveness for all LMH species = 96%). After controlling for the intended effects of the experimental treatments on dung density, the square-root-transformed data suggested unintended effects of the fences for only two LMH species (i.e., differences in activity levels among treatments that did not target those species; see Methods: Statistical Analysis). Warthog dung density was significantly greater in mesoherbivore-exclusion than megaherbivore-exclusion plots (HSD, *P*<0.01), neither of which differed significantly from open plots (*P*≥0.1); the significant treatment*site interaction (*F*_4,12_ = 3.5, *P* = 0.04) indicates that this effect was greatest at the low-rainfall site, and likely the result of a warthog that temporarily inhabited one of the mesoherbivore-exclusion plots (JRG and RMP, *pers. obs.*). Buffalo dung density was slightly but significantly greater in open plots than megaherbivore-exclusion plots (*F*_1,6_ = 8.1, *P = *0.03). This apparent reduction of buffalo activity in Mega relative to Open plots might actually reflect the activity of cattle, whose dung is difficult to distinguish from that of buffalo. Although herders are asked to keep cattle out of the plots, camera traps have recorded cattle within seven of the plots; such accidents may be more common in Open plots than in Mega plots, where the 2-m high fences offer a starker visual reminder to herdsmen than do the 1-m high posts surrounding Open plots. In any event, total dung deposition by buffalo/cattle is the lowest of all species (Fig. 2), and we do not believe that infrequent cattle incursions substantively affect our results.

Only two species showed significant variation in dung density across sites. Impala dung density was significantly greater in the low-rainfall site than in the intermediate- and high-rainfall sites (site: *F*_2,6_ = 15.0, *P* = 0.005; HSD, both pairwise *P*≤0.03). Zebra dung density was greater in the low-rainfall site than the intermediate-rainfall site (site: *F*_2,6_ = 6.1, *P* = 0.04; HSD, *P* = 0.03), neither of which differed significantly from the high-rainfall site. Dung-decomposition rates were analyzed using ANOVA with species, site, and their interaction as categorical factors, and with understory cover and sun exposure as ordinal effects. Rates differed among the three dominant LMH species tested (*F*_2,77_ = 21.5, *P*<0.0001; HSD, all pairwise *P*≤0.01). Mean percent decrease in weight of fresh dung over 30 d was greatest for dik-dik (88.8±0.03%, 12 of 30 piles disappeared completely), intermediate for impala (71.7±0.03%, 6 of 30 piles disappeared), and least for elephants (55±0.04%, 2 of 12 piles disappeared). Understory cover did not significantly affect decomposition rates (*F*_2,77_ = 0.54, *P* = 0.59), but sun exposure did (*F*_2,77_ = 4.37, *P* = 0.016), with significantly slower decomposition rates in full sun than beneath tree canopies (HSD, *P*<0.03). Decomposition rates did *not* differ significantly across sites (*F*_2,77_ = 0.71, *P* = 0.49) or show significant species*site interactions (*F*_4,77_ = 1.36, *P* = 0.25). We therefore conclude that our use of dung counts as an index of LMH activity levels is not likely to be biased by differential decay rates across sites.

Carnivores of all sizes (including lions, leopards, hyenas, wild dogs, and jackals) have been recorded in all treatments except total-exclusion, and leopards have been sighted repeatedly in total-exclusion plots, suggesting that the experimental barriers are more permeable to predators than to herbivores and that results are unlikely to be driven by predator exclusion.

### Abiotic Environment

**Figure 3:**
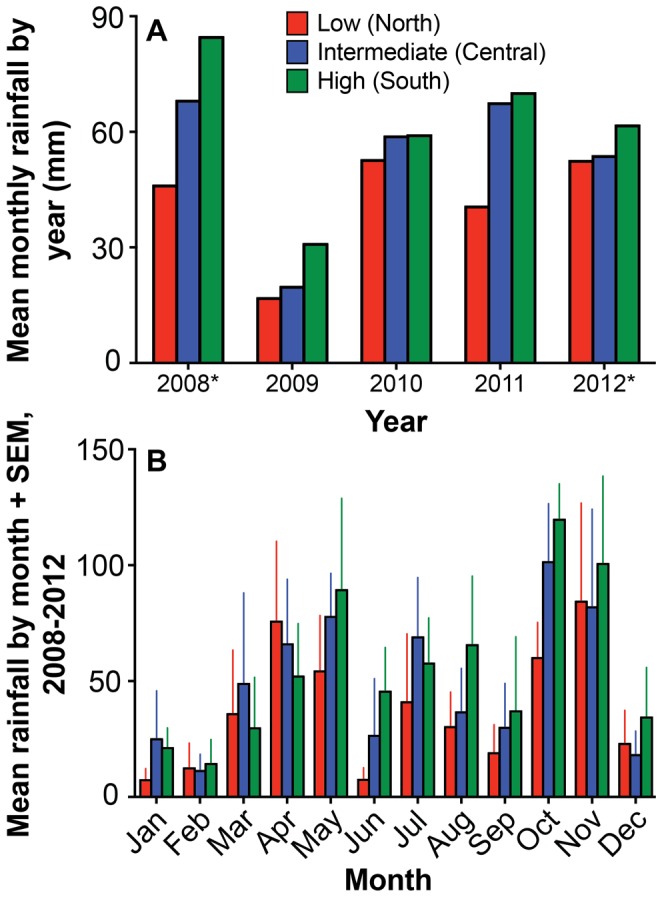
Mean monthly rainfall (A) within years and (B) across years at each of the three sites. The asterisks next to years 2008 and 2012 in the top panel indicate that data were available only for 3 and 5 months, respectively.

Following a drought in 2009, total annual rainfall at our high-rainfall sites was considerably greater in 2010 (710 mm) and 2011 (840 mm) than the 13-year average from a nearby rain gauge (641 mm). Across the three experimental sites, annual rainfall patterns since October 2008 have been consistent with expectations–greatest in the southern site, intermediate in the central site, and lowest in the northern site (Fig. 3a)–despite considerable month-to-month variability (Fig. 3b). The distribution of precipitation events across sites was more even: in the 629 days between 11 June 2010 (when we installed automated rain loggers) and 26 February 2012, the number of days with rainfall events was 152, 145, and 170 in the low-, intermediate-, and high-rainfall sites, respectively.

Soils are Alfisols in US Soil Taxonomy, with clay-enriched subsoils (argillic horizons) of high base saturation (∼100% in all profiles studied) (Fig. S3). The ustic moisture regime classifies them as Ustults, and in the absence of other diagnostic features (notably a kandic horizon) the soils at all exclosure sites classify as Typic Haplustalfs. This agrees with a previous soil-mapping exercise in the region [57], which classified soils developed on metamorphic basement rocks as Typic Haplustalfs or Typic Ustropepts (the latter no longer exist in Soil Taxonomy). Given the absence of information on moisture status at depth in this profile we did not consider the aridic/udic designations. We therefore consider our classifications to be preliminary and open to change in light of further analysis or new information (for example, on seasonal variation in subsoil moisture). Details of the soil profiles are provided in the Supporting Information.

There is little evidence of clay movement (e.g., clay films), but clay depletion in the upper horizons and enrichment at depth supports the designation of the subsoils as argillic. In the low- and intermediate-rainfall sites, the subsoils are extremely gravelly, with >90% gneiss fragments and bedrock at 0.86–1.34 m. The soils appear to be degraded through a legacy of overgrazing, particularly in the intermediate- and low-rainfall sites, with compacted surface horizons showing platy structure and extreme excavation resistance. This may impede root growth, but there are many fine and very-fine roots in the subsoil of all profiles. The presence of fine roots at depth is presumably because exchangeable base cation concentrations are highest in the subsoils, and indicates that analysis of surface soil alone may not adequately characterize nutrient status. All profiles have low aluminum saturation (≤2%) and low concentrations of organic matter (∼1%). The profile at the intermediate-rainfall site has a moderately acid surface soil (pH 5.8 in water), whereas the other profiles are slightly acid at the surface (pH 6.3–6.4). However, the high-rainfall site profile becomes strongly alkaline in the subsoil (up to pH 8.7 in water).

Several factors indicate a difference in the high-rainfall site profile compared to the low- and intermediate-rainfall site profiles. In particular, the high-rainfall site profile has (a) an absence of strong compaction/excavation resistance in the surface horizons, (b) an alkaline subsoil, and (c) much greater concentrations of exchangeable base cations and a higher effective cation exchange capacity throughout the profile. Based on the proximity of the high-rainfall (southern) exclosures to the phonolite scarp face, above which soils are clay-rich Vertisols with alkaline subsoils containing carbonate nodules, it seems likely that the area around these exclosures has received considerable input of material from the escarpment, either during the original emplacement of the phonolite lava flows, or subsequently via runoff or dust deposition. (This may in turn explain the presence of *Acacia drepanolobium* at the high-rainfall site; this species dominates the tree community on the plateau Vertisols.).

Surface soils collected in 2009, five months into the experiment, had significantly lower mean pH in the intermediate-rainfall site than in the high- or low-rainfall sites (Table 1; see also raw data in Table S2). Concentrations of exchangeable Ca, Mg, K, extractable NO_3_, NH_4_, and P, and total percent N and C did not differ across sites (all *P*>0.09), and no soil attribute differed significantly by treatment or showed a significant treatment*site interaction (all *P*>0.07).

**Table 1:** Surface-soil attributes showing significant variation across treatments and/or sites.

			HSD, Treatment			HSD, Site			
Attribute	Year measured	Treatment *P*	Total	Open	Site *P*	High	Int.	Low	Treat×Site
pH	2009	0.07	–	–	**0.001**	A	B	A	0.50
pH	2012	0.73	–	–	**0.003**	A	B	A	0.59
% Sand	2012	0.40	–	–	**0.05**	A	A	A	0.13
% Clay	2012	0.96	–	–	**0.05**	A,B	A	B	0.61
% Silt	2012	**0.01**	A	B	**0.009**	A	B	B	**0.004**
Ca (ppm)	2012	**0.007**	A	B	0.18	–	–	–	**0.015**
S (ppm)	2012	0.84	–	–	**0.04**	B	A	A,B	0.21
K (ppm)	2012	0.23	–	–	0.36	–	–	–	**0.0004**
Al (ppm)	2012	0.86	–	–	**0.0009**	B	A	C	0.93

**Notes:** Degrees of freedom in 2009 = 3,18 for treatment; 2,6 for site; and 6,18 for treatment*site. In 2010 and 2012, df = 1,6 and 2,6 for treatment and site, respectively, and 2,6 for treatment*site. Levels of treatment and site that do not share the same letter were significantly different in Tukey’s HSD post-hoc tests; the level with the highest mean is represented by letter A, the next highest by letter B, etc.

Surface soils collected from total-exclusion and open plots in 2010 did not differ significantly across any of the fixed effects for any of the variables measured (NO_3_, NH_4_, percent sand, silt, and clay; all *P*>0.1). Inspection of the data, however, revealed that one block in the intermediate-rainfall site had a disproportionately high clay:sand ratio (Fig. S4a).

This outlying value was confirmed in analyses of surface soils collected in 2012. In that year, mean clay content was significantly greater in the intermediate-rainfall site (30.1±3.0%) than in the low-rainfall site (18.1±0.8%; *F*_2,6_ = 5.1, *P = *0.05; HSD *P = *0.04; Table 1). The effect of site was also significant in the mmANOVA for percent sand content (*F*_2,6_ = 5.3, *P = *0.05), with higher values in the low-rainfall site than the other two sites (HSD, *P* = 0.06 and 0.08 for comparisons between low-rainfall sites and intermediate- and high-rainfall sites, respectively; Table 1). Finally, percent silt was significantly greater in the high-rainfall site (19.5±1.4%) than either the intermediate- (14.4±0.9) or low-rainfall (13.4±0.6) sites (*F*_2,6_ = 11.4, *P = *0.009; HSD, both *P*≤0.02; Table 1). Plots did not cluster strongly by site or treatment when the 2012 soil data were analyzed using MDS (Fig. S4b), although the effect of site (alone) was significant in the corresponding perMANOVA analysis (*F*_2,12_ = 3.9, *P* = 0.01; treatment: *F*_1,12_ = 0.7, *P* = 0.52; site*treatment: *F*_2,12_ = 1.6, *P* = 0.20), reflecting differences between the high- and intermediate-rainfall sites.

The 2012 data corroborated the 2009 result of lower pH in the intermediate-rainfall site (mean of 2009 and 2012 measurements: 5.24±0.11) relative to low- (5.99±0.09) and high-rainfall (6.19±0.05) sites. Several additional surface-soil properties differed significantly across sites and/or treatments in the 2012 samples (Table 1, Table S2). Only two soil attributes differed significantly across treatments: calcium concentrations and percent silt were both greater in total-exclusion than in open plots (1356±130 vs. 1146±91 mg/kg for Ca; 16.70±1.49 vs. 14.83±0.81 for percent silt), but these significant main effects of treatment were driven by the high-rainfall site (treatment*site interactions in Table 1).

Mean infiltration rates varied little across sites, whether we used data from all 14 ring fillings (1.67–2.38 mm/min from high to low rainfall, respectively) or from only the last five (1.40–2.02 mm/min, respectively). Variation was even less across treatments (ranging from 1.72–2.17 and 1.52–1.82 mm/min, for all 14 fillings and for the last five, respectively). Neither site, treatment, nor their interaction were statistically significant predictors of infiltration rates in mmANOVA, regardless of how the data were truncated.

### Proxies for Primary Productivity

**Figure 4:**
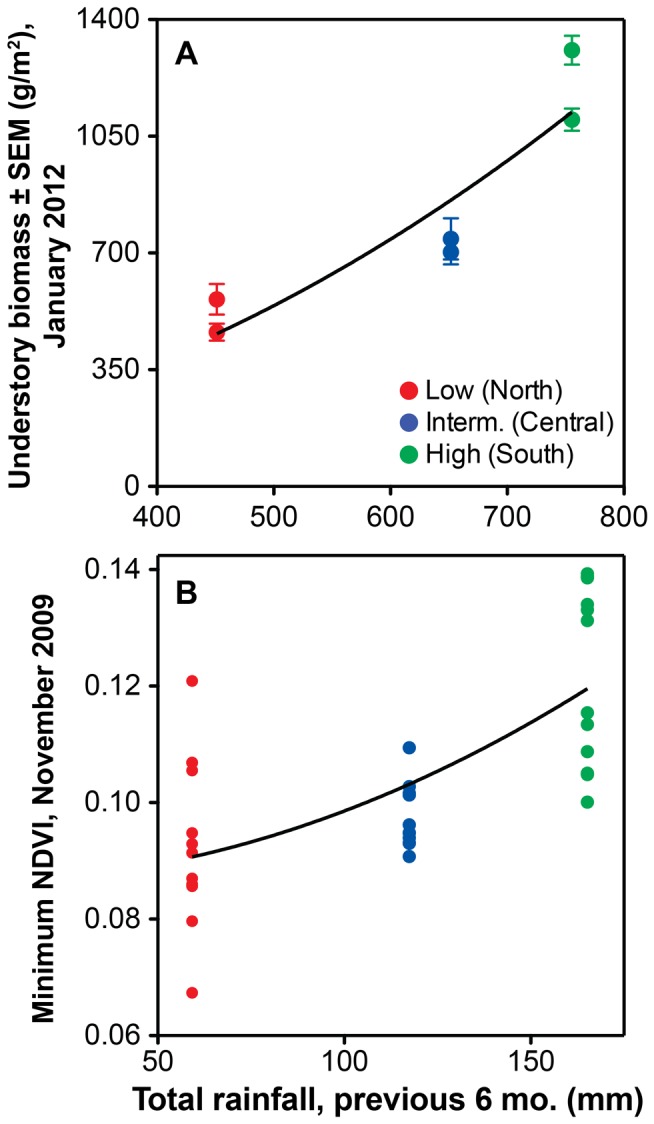
Relationship between two metrics of productivity and rainfall in the six months prior to productivity measurement. (A) Mean peak understory biomass (grasses and forbs) in two 64-m^2^ grids located within total-exclusion plots at each site. (B) Mean NDVI in each plot, calculated from Quickbird satellite imagery. Rainfall was squared in regression analyses to better fit the data.

Mean understory biomass density within total-exclusion plots increased from low- (512±49 g/m^2^) to intermediate- (722±20 g/m^2^) to high-rainfall (1204±104 g/m^2^) sites as a function of squared rainfall over the six months prior to harvesting (*r* = 0.92, *F*_1,4_ = 21.5, P<0.01; Fig. 4a). In the one-way comparison across sites (whole-model *F*_2,3_ = 27.7, *P* = 0.01), high-rainfall sites had greater biomass density than intermediate- and low-rainfall sites (HSD, *P = *0.03 and 0.01, respectively); the latter two did not differ significantly from each other (HSD, *P* = 0.2).

Mean NDVI likewise differed across sites (*F*_2,18_ = 9.2, *P = *0.015), again being greater in high-rainfall sites (0.33±0.01) than in either low- (0.25±0.02; HSD *P = *0.056) or intermediate-rainfall sites (0.22±0.01; HSD *P* = 0.01), which again did not differ significantly from each other. Mean NDVI also differed across treatments (*F*_3,18_ = 5.8, *P = *0.006), being significantly greater in total- and mesoherbivore-exclusion plots (0.29±0.02 and 0.28±0.02, respectively) than in open plots (0.24±0.02; HSD *P*<0.035), and marginally greater in total- than in megaherbivore-exclusion plots (0.26±0.02; HSD *P* = 0.066). The minimum (Fig. 4b), maximum, and mean NDVI values for each plot were all positively correlated with rainfall-squared over the six months prior to the collection of the Quickbird imagery (*r* = 0.45–0.69, all *F*_1,34_≥8.6, all *P*≤0.006).

### Understory Vegetation

**Figure 5:**
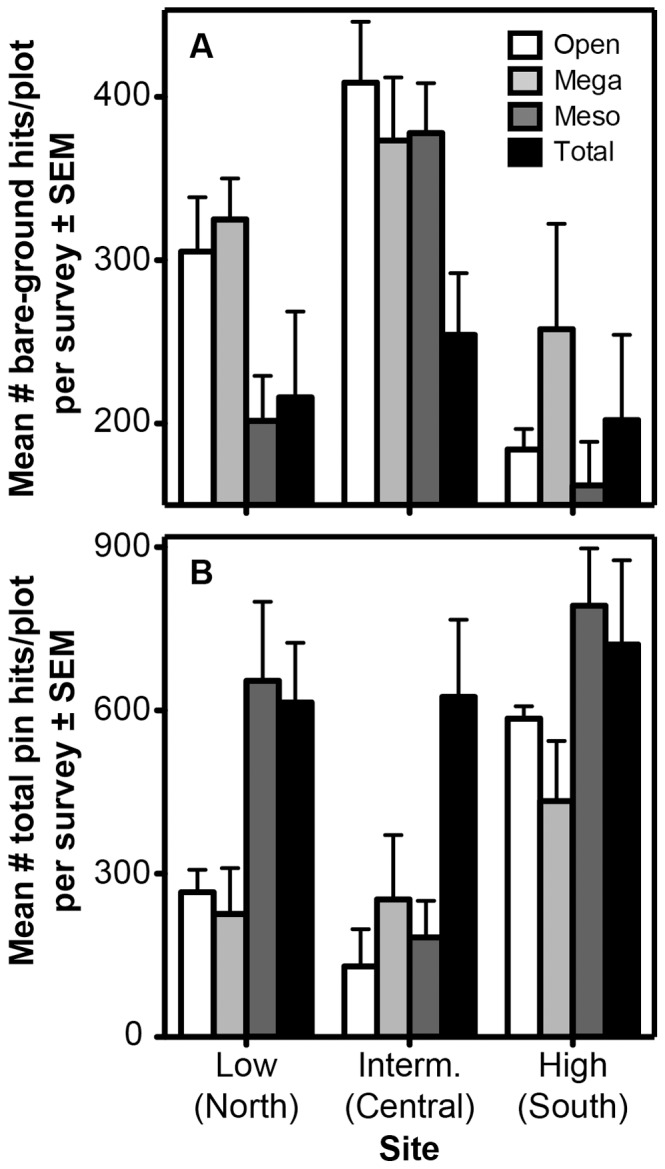
Trends in (A) extent of bare ground and (B) total understory vegetation pin hits across sites and treatments. Data are out of 490 total pin drops per plot per survey.

When we averaged data for each plot across the seven understory surveys from 2008–2011, the mean number of bare-ground pin hits decreased from intermediate-rainfall (72% of pin drops) to low-rainfall (53%) to high-rainfall sites (41%; *F*_2,6_ = 8.4, *P* = 0.02; intermediate significantly greater than high sites, HSD *P* = 0.015), and was greater on average in open and megaherbivore-exclusion plots (61% and 65% respectively) than in mesoherbivore- and total-exclusion plots (50% and 45% respectively; treatment: *F*_3,18_ = 5.3, *P* = 0.009; HSD *P = *0.01 and 0.055 for the Mega-Total and Open-Total comparisons, respectively) (Fig. 5a). When each survey was analyzed independently, site effects were significant in all surveys but the first; the typical pattern was for bare-ground pin hits to be significantly more frequent in the intermediate- than the high-rainfall site, with middling values at the low-rainfall site. Treatment effects on the frequency of bare ground were significant only in the four surveys conducted in 2009 and 2011 (and thus were not driven by season), with the rank ordering of treatments in these comparisons being Mega ≥ Open ≥ Meso ≥ Total.

Overall understory density (sum of total vegetation pin hits per plot, averaged across seven surveys) was significantly greater in high- than in intermediate-rainfall sites (1.3±0.1 vs. 0.6±0.15 hits per pin; HSD, *P* = 0.04), and in total- and mesoherbivore-exclusion plots (1.3±0.1 and 1.1±0.2 hits per pin, respectively) relative to megaherbivore-exclusion and open plots (0.6±0.1 and 0.7±0.1, respectively; HSD, pairwise *P*<0.03; Fig. 5b). The significant treatment*site term in this model (*F*_6,18_ = 2.7, *P* = 0.046) reflects a disproportionately large difference between total- and mesoherbivore-exclusion plots at the intermediate-rainfall site (Fig. 5b). When we analyzed each survey separately (square-root transformed data), the effect of site was significant in four of the seven surveys (February 2009, October 2010, February 2011, and October 2011). In three of these, understory density was significantly greater in the high-rainfall site than the intermediate-rainfall site, neither of which differed significantly from the low-rainfall site; in the fourth (February 2011), understory density was greater in the high-rainfall site than both low- and intermediate-rainfall sites. In terms of treatment, understory density was always greatest in Total plots and second-greatest in Meso plots; the difference between these two treatments was statistically significant in only one of the surveys (October 2009, at the tail end of a yearlong drought; HSD, *P* = 0.003). Open and Mega plots, which together had the lowest understory densities, were statistically indistinguishable from each other in all surveys. The most frequent pattern (obtained in four of the surveys) was for Total plots to have significantly greater understory density than Open and Mega plots, with no significant differences between Meso plots and any of the other treatments (HSD pairwise comparisons).

Rank-abundance curves showed that understory communities were dominated by the same three grass species at each site: *Pennisetum stramineum*, *Cynodon plectostachyus*, and *C. dactylon* (collectively accounting for 55%, 51%, and 50% of total pin hits at low-, intermediate-, and high-rainfall sites, respectively; Fig. S5). Of the 10 most abundant species at each site, six grasses were shared across all sites (the three above, plus *Microchloa kunthii*, *Eragrostis tenuifolia*, and *Aristida* sp.).

When we analyzed pin-hit data for individual species (averaged across seven surveys and square-root transformed), the treatment effect was significant for 13 species (Table 2), including the three numerically dominant species. These species were least common in either Open or Mega plots in all but one case (*Heteropogon contortus*, lowest in Total, although this effect was pronounced only at the high-rainfall site), and were most common in either Total or Meso plots in all but two cases (*Cyperus* sp. and *H. contortus*, highest in Open). The effect of site was significant for 27 species; 10 of these were most common in the low-rainfall site, two in the intermediate-rainfall site, and 15 in the high-rainfall site (Table S3). Twenty-three species showed no significant effects of treatment, site, or their interaction. An additional 58 species for which we recorded <100 pin hits (corresponding to <0.081% cover) were deemed too rare to justify a statistical comparison.

**Table 2:** Understory plant species showing significant variation across treatments.

								HSD, Treatment			HSD, Site		
Species	Overall percent cover		Overall rank abundance	Treat-ment *P*	Total	Meso	Mega	Open	Site *P*	High	Int.	Low	Treat×Site
*Cynodon plectostachyus*	18.8		1	**0.0034**	A	A,B	B	B	**0.0088**	A	B	A	0.70
*Pennisetum stramineum*	16.5		2	**0.028**	A	A,B	A,B	B	0.35	–	–	–	0.06
*Cynodon dactylon*	14.7		3	**0.01**	A	A,B	B	B	0.75	–	–	–	0.10
*Plectranthus* sp. “small”	2.0		9	**0.01**	A	A,B	A,B	B	**0.02**	A,B	A	B	**0.003**
*Brachiaria leersioides*	1.8		10	**0.03**	A,B	A	A,B	B	**0.002**	B	B	A	0.27
*Commelina* sp.	1.2		13	**0.009**	A	A,B	A,B	B	**0.047**	A	A	A	0.13
*Indigofera* sp. “small”	0.90		15	**0.004**	A	A,B	B	B	0.40	–	–	–	0.19
*Cenchrus ciliaris*	0.61		23	**0.035**	A,B	A	B	A,B	0.07	–	–	–	**0.04**
*Ipomoea biflora* (syn. *sinensis*)	0.42		27	**0.0001**	A	B	C	B,C	**0.0035**	A	B	A,B	0.11
*Cyperus* sp.	0.38		30	**0.018**	A,B	A,B	B	A	**0.0025**	A	B	A	0.06
*Heteropogon contortus*	0.30		33	**0.05**	A	A	A	A	**0.04**	A	B	A,B	**0.01**
*Solanum campylacanthum*	0.27		38	**0.04**	A,B	A	A,B	B	0.38	–	–	–	0.18
*Helichrysum glumaceum*	0.17		47	**0.02**	A	A	A	A	0.27	–	–	–	0.37

**Notes:** Degrees of freedom = 3,18 for treatment; 2,18 for site; and 6,18 for treatment*site. Levels of treatment and site that do not share the same letter were significantly different in Tukey’s HSD post-hoc tests; the level with the highest mean is represented by letter A, the next highest by letter B, etc. Percent cover is the number of pin hits for each species divided by the total number of pin drops (123,480) in the experiment to date. Data for *Indigofera* sp. “small” are from only the most recent three surveys, as opposed to seven surveys for other species. Results for the remaining understory plant species are shown in Table S3.

The impact of herbivory differed significantly across sites for only four understory species (see treatment*site interaction in Tables 2 and S3). Three of these were suppressed by LMH, but only at low-rainfall (*Plectranthus* sp. and *Sida* sp.) or intermediate-rainfall sites (*Cenchrus ciliaris*). The fourth species, *Heteropogon contortus*, was apparently facilitated by LMH (as noted above), but only at the high-rainfall site.

Asymptotic species richness (Chao2, calculated for each survey and then averaged across all surveys) was greater in the high-rainfall site (50.5±1.9 species/plot) than in the intermediate- (39.5±2.0; *F*_2,6_ = 31.4, *P = *0.0007; HSD, *P* = 0.008) and low-rainfall sites (43.9±2.6; HSD, *P* = 0.068), with no significant difference between low- and intermediate-rainfall sites; the effect of treatment was non-significant (*F*_3,18_ = 1.96, *P* = 0.16). When understory species richness was analyzed for each survey independently, site effects typically followed the same pattern described above, and the treatment effect was significant only in the second survey (February 2009; *F*_3,17_ = 4.8, *P* = 0.01), when species richness was greater in total-exclusion plots (48.2±3.9 species/plot) than in both megaherbivore-exclusion and open plots (37.1±4.0 and 34.2±3.6, respectively; HSD, *P* = 0.04 and 0.01, respectively). The treatment*site interaction for species richness was not significant in any survey.

Similarly, understory Shannon diversity (averaged across all surveys) was 23% greater in high- than intermediate-rainfall sites (*F*_2,6_ = 6.56, *P* = 0.03; HSD, *P* = 0.027), with no significant effect of treatment (*F*_3,18_ = 0.3, *P* = 0.8). The same patterns in diversity were observed consistently when each survey was analyzed independently. In contrast, Shannon evenness (averaged across all surveys) did not show significant effects of site (F_2,6_ = 0.4, *P* = 0.68), but did appear to differ across treatments (*F*_3,18_ = 2.93, *P* = 0.06): open plots had the greatest evenness and total-exclusion plots the lowest, and this contrast approached statistical significance (HSD, *P* = 0.06). However, when evenness was analyzed for each survey separately, this same pattern was only observed in February 2010 (treatment: *F*_3,18_ = 4.0, *P* = 0.02; HSD contrast of open vs. total-exclusion plot, *P* = 0.03).

**Figure 6:**
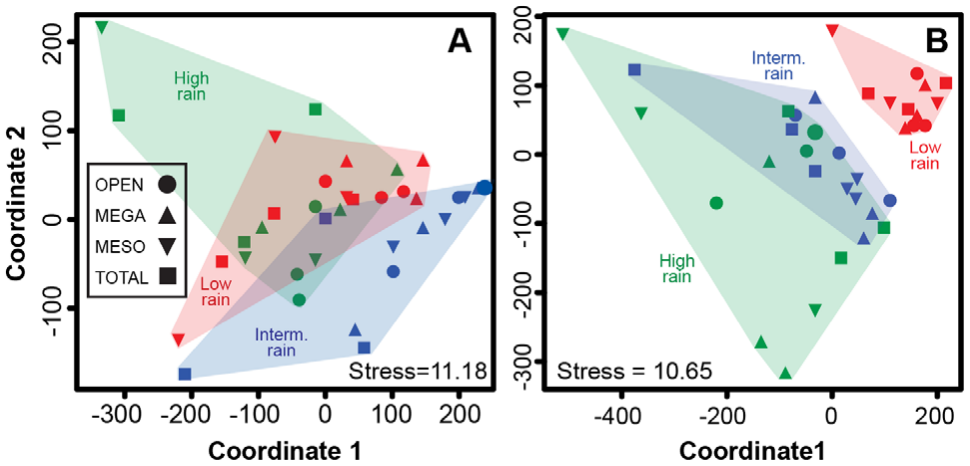
Non-metric multidimensional scaling plots illustrating patterns of community similarity across sites and treatments for (A) understory and (B) overstory plants.

Community similarity of all 36 plots showed no strong clustering in understory species composition by site, treatment, or block within site (MDS, Fig. 6a), although intermediate-rainfall sites tended to be distinct, and the Meso and Total plots in one block of the high-rainfall site were outliers. The corresponding perMANOVA analysis indicated significant differences in community similarity across both sites (*F*_2,24_ = 8.1, *P*<0.0001) and treatments (*F*_3,24_ = 2.6, *P* = 0.008), with no significant interaction term (*F*_2,24_ = 1.2, *P* = 0.2).

### Overstory Vegetation

Overall overstory density in 2012 (total stems of all species, square-root transformed) increased with increasing rainfall (site: *F*_2,6_ = 23.1, *P* = 0.0015) and was significantly greater in the high-rainfall site (2093±243 individuals/ha) than the intermediate (1260±219 individuals/ha; HSD, *P* = 0.008) and low-rainfall sites (923±91 individuals/ha; HSD, *P* = 0.001). There was no significant main effect of treatment on overall overstory density across treatments (*F*_3,18_ = 1.5, *P* = 0.25), although a significant treatment*site interaction (*F*_6,18_ = 2.7, *P* = 0.045) suggested that woody density was greater in Total plots than other treatments at low- and intermediate-rainfall sites only. We analyzed densities separately (square-root transformed data) for the seven common woody species that occurred at all sites. Three of these showed significant effects of treatment. *Acacia etbaica* densities were greater in Mega than in Open plots (treatment: *F*_3,18_ = 3.5, *P* = 0.037; HSD *P* = 0.03), but only at the high-rainfall site (in part because this species is rare at the low-rainfall site; interaction: *F*_6,18_ = 4.3, *P* = 0.007). *Acacia mellifera* densities were significantly greater in Total plots (treatment: *F*_3,18_ = 6.0, *P* = 0.005) than in Meso (HSD, *P* = 0.02) and Open plots (HSD, *P* = 0.004). Finally, *Balanites aegyptiaca* densities were significantly greater in Total than in Open plots (treatment: *F*_3,18_ = 4.2, *P* = 0.02; HSD *P* = 0.016). *Acacia brevispica*, *Croton dichogamus*, *Grewia* sp., and *Boscia angustifolia*, did not differ significantly across treatments (all *P*>0.07).

We recorded at least 27 overstory species in 2012, and their densities differed across sites (Fig. S6). Four species (*A. brevispica*, *A. mellifera*, *A. etbaica*, and *Croton dichogamus*) were among the seven most-abundant taxa at every site; *A. brevispica* was first- or second-most abundant at all sites, and *A. etbaica* was the most abundant species at the intermediate- and high-rainfall sites. Community similarity of overstory vegetation was driven by site, with considerable overlap between intermediate- and high-rainfall sites and no overlap between low-rainfall sites and the other two (Fig. 6b), due in part to the relative rarity of *A. etbaica* at the low-rainfall site and to two succulent *Euphorbia* species that were common at the low-rainfall site and absent from the others (Fig. S6). Analysis with perMANOVA corroborated the strong dissimilarity in composition across sites (*F*_2,24_ = 16.74, *P*<0.0001), with no significant effects of treatment (*F*_3,24_ = 1.2, *P* = 0.26) or treatment*site (*F*_6,24_ = 1.3, *P* = 0.17).

**Figure 7:**
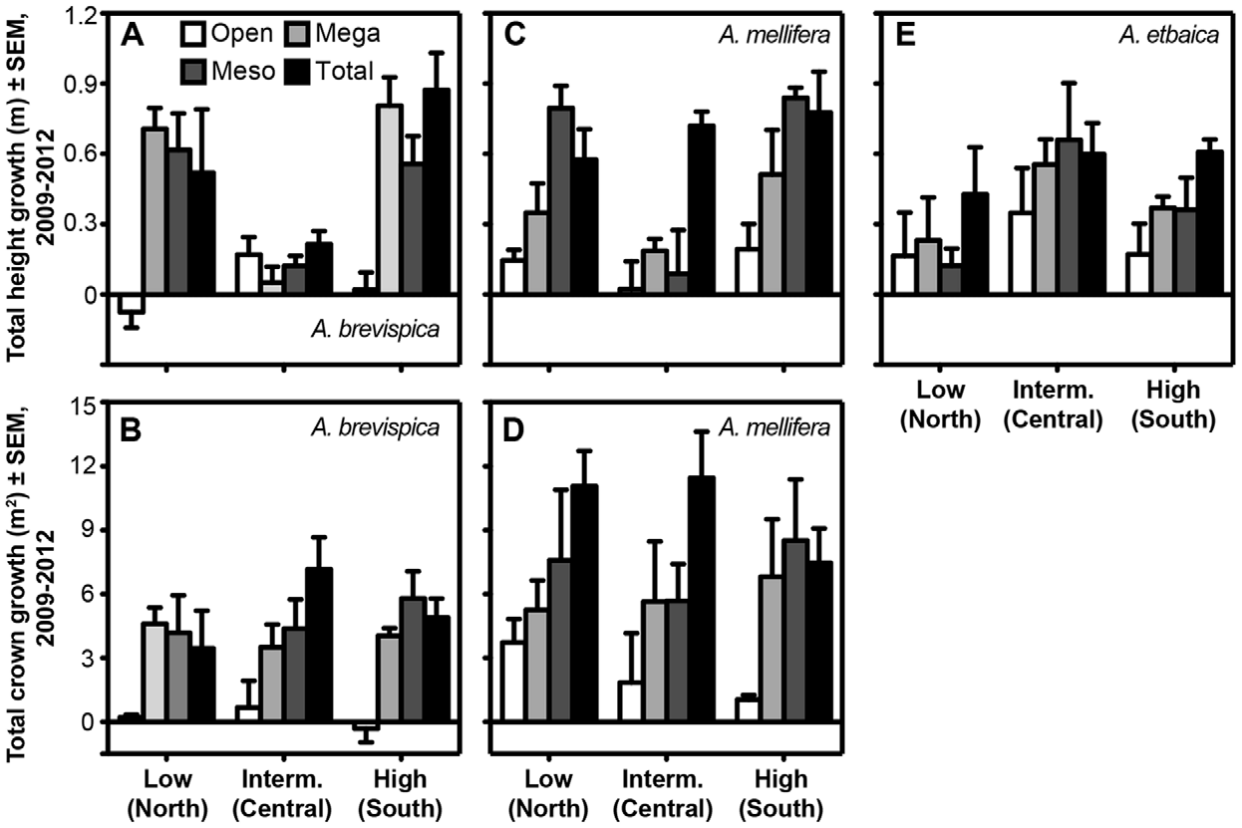
Rate of growth in height (A, C, E) and canopy breadth (B, D) for the three dominant *Acacia* species across sites and treatments.

Both height and canopy growth of *A. brevispica* were significantly greater in all three exclusion treatments than in open plots (Fig. 7a,b; *F*_3,18_ = 12.1, *P* = 0.0001 for height; *F*_3,18_ = 11.0, *P* = 0.0002 for canopy area; HSD, *P*<0.005 for all pairwise comparisons involving open plots). A significant treatment*site interaction in the model for *A. brevispica* height growth (F_6,18_ = 4.3, *P = *0.008) reflected the lack of significant treatment effects at the intermediate-rainfall site (Fig. 7a). Mean *A. mellifera* growth rates increased with each successive reduction in the LMH community (Fig. 7c,d), suggesting that browsers across the body-size spectrum influence growth rates in this species; however, the relative impact of different LMH groups varied across sites, especially for height growth (treatment*site *F*_6,18_ = 2.53, *P* = 0.059). For both *A. brevispica* and *A. mellifera*, mean height-growth rates were significantly greater in the high-rainfall site than the intermediate-rainfall site, but site did not affect canopy growth rates for either species. For *A. etbaica*, height growth (only) was greater in total-exclusion than in open plots (HSD, *P* = 0.01; Fig. 7e), with middling (and virtually identical) values in mega- and mesoherbivore-exclusion plots, and with no significant site or treatment*site effects. Mean height growth of *Balanites aegyptiaca* was greater in the three exclusion plots (range: 0.39–0.53 m) than open plots (−0.36 m), but statistical power was low because this species occurred in only 22 of 36 plots (treatment: *F*_3,8.5_ = 3.1, *P* = 0.087). *Croton dichogamus* growth rates were also lowest on average in open plots, but not significantly so.

### Small-mammal Community

**Figure 8:**
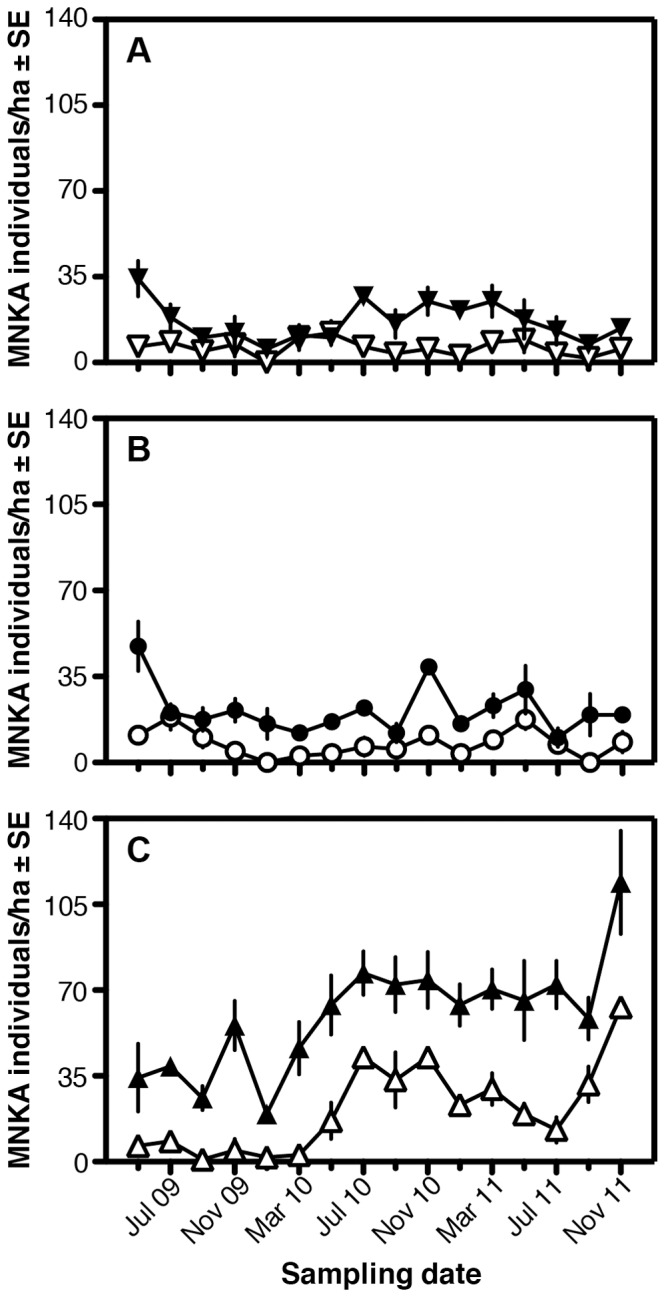
Temporal dynamics in the minimum number known alive of all small mammals in total-exclusion (filled markers) and open plots (open markers) at (A) low-rainfall, (B) intermediate-rainfall, and (C) high-rainfall sites.

From May 2009 to November 2011, we recorded 1789 unique individuals of at least 16 species of small mammals in 56,448 trap-nights over 16 sampling bouts (Table S1). Small-mammal communities were dominated by five taxa–*Gerbilliscus robustus*, *Mus* spp., *Aethomys hindei*, *Acomys kempi*, and *Saccostomus mearnsi*–collectively accounting for >80% of captured individuals. Two taxa (*G. robustus* and *Mus* spp.) were nearly ubiquitous, being recorded from at least one plot in every site in 15 of the 16 sampling bouts. Three taxa (*Acomys percivali*, *Dendromus* sp., and *Grammomys dolichurus*) have been captured only once to date. Mean MNKA densities (all species combined, averaged for each plot over all sampling bouts) differed significantly across sites (Fig. 8; *F*_2,6_ = 51.1, *P* = 0.0002), being roughly three-times greater in high-rainfall sites (44.4±9.3 individuals/ha) than intermediate (14.5±3.2; HSD, *P* = 0.0004) or low-rainfall sites (11.4±2.6; HSD, *P* = 0.0002); the latter two sites did not differ significantly (HSD, *P* = 0.6). Overall, mean densities in Total plots (32.5±7.2) were nearly triple those in Open plots (11.6±2.5; *F*_1,6_ = 42.9, *P* = 0.0006). Finally, there was a significant treatment*site interaction (*F*_2,6_ = 7.4, *P* = 0.02), with significantly greater densities in high-rainfall exclusion plots than in any other site-treatment combination (Fig. 8; HSD, all pairwise *P*<0.004).

## Discussion

### Validity of the Experimental Design

Our results show that the UHURU treatments function as intended. The three exclosure treatments reduced dung deposition of targeted herbivore species by ∼96% overall. Moreover, the actual impact of incursions within these treatments is likely less than the dung-count failure rate implies. On the infrequent occasions when impala and zebra have breached the mesoherbivore and total-exclusion plots, our observations indicate that they do not feed, but rather pace the perimeter of the fence until they escape or are released. Juvenile elephants occasionally stray beneath the megaherbivore-exclusion fences while the herd feeds nearby, but these individuals are small and they do not stray far from the group. Finally, heavy rains sometimes carry dung pellets of giraffe, antelope, and zebra into adjoining plots, contributing to an overestimate of treatment failure rate.

As mentioned above, the elevated dung deposition of warthogs in Meso relative to Mega plots (Fig. 2) likely resulted from the temporary residence of a warthog in one of the low-rainfall Meso plots; we suspect that this result was idiosyncratic and will not persist. As noted in the Results, the apparent reduction of buffalo dung in Mega relative to Open plots might actually reflect the activity of cattle, whose dung is difficult to distinguish from that of buffalo.

Effects of site on dung density were significant for only two species, impala and zebra, and indicated that activity of both species was greatest at the low-rainfall site. This is surprising in light of the expectation [52], [80] that local herbivore abundance should be positively correlated with rainfall and primary productivity. At our sites, human activity increases along the rainfall gradient from low to high, so the site effects for impala and zebra might in part reflect avoidance of people. The absence of a more general correlation is encouraging, although the apparently greater activity levels of two common mesoherbivores at one site suggests the need to intensify LMH monitoring as the experiment proceeds, so that we can account statistically for any biases if necessary.

Rainfall, the second major factor in our experimental design, also followed the anticipated pattern over the first three full years of the study (Fig. 3). Not surprisingly, there is variation in the magnitude of the monthly and yearly differences in water input across sites. However, the data suggest that differences in rainfall across sites are primarily a function of the quantity of rainfall per event, rather than the frequency of rainfall events, which is similar at all sites.

Most soil attributes did not differ significantly across sites or treatments. All three sites were classified as Typic Haplustalfs (Alfisols). This generally supports one assumption of our experimental design–that comparisons of LMH effects across sites will not be heavily influenced by abiotic variables other than rainfall. Collectively, analyses of surface soils collected in 2009 and 2010 revealed significant variation across sites in only one soil attribute (pH). In 2012, the intermediate-rainfall sites had the lowest surface-soil pH (as observed in 2009) and the highest % clay, sulfur, and aluminum contents (but see soil-profile data). Nevertheless, points did not cluster strongly by site in MDS (Fig. S4b). The only apparent cluster in that graph comprised the high-rainfall total-exclusion plots, which is consistent with the significant treatment*site interactions for silt, calcium, and potassium concentrations (Table 1), all of which had disproportionately high values in the high-rainfall total-exclusion plots. The reason for the pronounced effects of exclusion on these three variables at high-rainfall sites is not yet known, and neither is the cause of discrepancies in the results for soil texture in samples taken in 2010 and 2012 (which were collected using slightly different strategies and analyzed by different labs). Future sampling using standardized methods will reveal whether these results are persistent.

There is a potentially important caveat to our conclusion that soil composition is generally similar across sites. The inter-annual mean surface-soil pH of 5.24 found at intermediate-rainfall sites is considered strongly acidic [81], whereas those the low- and high-rainfall sites are considered only moderately-to-slightly acidic. Aluminum toxicity can be a problem in acidic soils [82], and the threshold pH above which Al becomes insoluble in many soils is approximately 5.2 [83]. It is therefore noteworthy that Al concentrations were also significantly greater at intermediate-rainfall when measured in 2012. However, Al saturation was extremely low to depth in the three profile pits (≤2%), so it seems unlikely that Al toxicity contributed to the lower-than-expected peak-biomass and NDVI measurements in the intermediate-rainfall site (Fig. 4), or to other differences in plant community composition in that site relative to the other two (Figs. 5, 7; Tables 2, S2). Likewise, P deficiency, another problem associated with acidic soils, is unlikely given the relatively high Mehlich-extractable (plant-available) P concentrations in surface soils. Surface horizons at the central and northern sites appear to become extremely hard during dry periods, which can impede root growth or kill existing roots, although fine roots were abundant to bedrock in all profiles studied. Nonetheless, potential edaphic constraints on plant growth warrant further investigation.

Another important assumption of our design is that variation in rainfall across sites translates into measurable differences in primary productivity, since we expect productivity (rather than rainfall per se) to modulate the direct and indirect effects of LMH. Both field- and satellite-derived proxies for productivity increased across sites as a function of recent rainfall (Fig. 4) and were greatest overall in the high-rainfall site. Neither proxy, however, differed significantly between intermediate- and low-rainfall sites, despite the fact that total precipitation in the intermediate-rainfall site was closer to that in the high-rainfall site than the low-rainfall site (Fig. 3). We have already discussed soil pH as a possible factor limiting production at the intermediate-rainfall site. Another factor might be a legacy of intensive cattle grazing at this site prior to 2007 (Mike Littlewood, Mpala Ranch, personal communication). This site currently has extensive areas of hard, bare soil with high surface clay content, which appear resistant to colonization by plants. These features might contribute to high runoff rates and a decoupling of landscape-scale production from rainfall. Our measurements of infiltration capacity did not reveal any consistent differences across sites; however, these trials were only performed at one location within each plot (equating to 12 locations within each site). Increased spatial and temporal replication of these measurements will help to elucidate the lower-than-expected production:rainfall ratio in the intermediate-rainfall site, as will an evaluation of the potential effects of soil acidity. Likewise, it will be helpful to refine and increase the spatiotemporal replication of our productivity measurements, given the spatial heterogeneity of vegetation cover and the difficulty of accurately measuring primary productivity at large scales.

### Effects of Herbivory and Environmental Context on Response Variables

Not surprisingly, understory density was greatest (and coverage of bare ground was least) in plots accessible to the fewest LMH species. In general, the treatments segregated into two groups: high understory density in total- and mesoherbivore-exclusion plots and low density in open and megaherbivore-exclusion plots. Open and Mega plots were never significantly different, and Meso and Total plots differed in only one of seven surveys. This suggests that mesoherbivores strongly regulate total understory density, that warthogs and dik-dik have considerably weaker effects, and that megaherbivores have no detectable effect (in this case, elephants, since giraffes rarely browse the understory [84]). The mesoherbivore size class is the most species rich, comprising eight species recorded in our plots, and also the most functionally diverse, ranging from strict grazers (zebra, buffalo) to strict browsers (gerenuk). Moreover, impala are abundant at our sites and consume a broad range of both grasses and forbs [85]–[87], and dung counts suggest that they are particularly active at the low-rainfall site, where strong effects of mesoherbivore-exclusion were observed (Fig. 5). All of these factors likely contributed to the pronounced effects of mesoherbivore exclusion, although the limited impact of dik-dik, warthog, and elephants is still noteworthy.

Almost all of the individual understory species that differed significantly across treatments were most abundant in total- or mesoherbivore-exclusion plots (Table 2). If competition were a major limiting force (or if overcompensation by plants following herbivory were a frequent occurrence), then we might expect some species to be more abundant in open and megaherbivore-exclusion plots than in total and mesoherbivore exclosures (where overall understory densities were higher). However, this pattern was observed for only one species, *Heteropogon contortus*. It is possible, though, that more species will show this pattern in the future, following a longer history of exclusion.

The observed trends in understory abundance across sites–with 15 species most common at high-rainfall sites, 10 at low-rainfall sites, and only two at intermediate-rainfall sites–are consistent with a plant species pool containing both mesic- and xeric-adapted species. Understory species richness and diversity were both greatest in high-rainfall sites and did not differ significantly across treatments, perhaps due to the relatively short duration of the experiment to date. In contrast, community evenness did not differ significantly across sites, but was greater in open than in total-exclusion plots, suggesting that the proportional representation of competitively dominant plant species increases in the absence of LMH. In time, this discrepancy in evenness may lead to treatment effects in species richness via competitive exclusion; that we did not observe such effects in the present dataset may reflect a combination of low statistical power and limited effect sizes after only three years of the experiment. Theory predicts that competitive exclusion following LMH exclusion should be most likely in resource-rich areas (our high-rainfall site) [53]. Continued monitoring should enable us to test this prediction.

Trends in woody-stem density paralleled trends in rainfall across sites, as expected. The lack of significant treatment effects on overall woody density is perhaps not surprising after only 3.5 years: seedling and sapling recruitment in savannas tends to be infrequent and episodic [43], [88], [89], and density responses might take more time to materialize. However, the significant effects of exclusion on three of the most common overstory species corroborate previous work showing that LMH (and megaherbivores in particular) regulate shrub dynamics in this system [60], and suggest that treatment effects on total stem density are likely to materialize eventually.

*Acacia brevispica* growth rates were regulated by megaherbivores (Fig. 7a,b). The absence of a significant megaherbivore effect on height at the intermediate-rainfall site (Fig. 7a) could be caused by either low abundance or differential diet selection of elephants at that site, or by an interaction between herbivory and soil attributes. We consider the latter most likely: our dung counts do not suggest consistent differences in elephant activity across sites, and megaherbivore exclusion *did* reduce *A. brevispica* canopy growth (Fig. 7b)–as well as the height and canopy growth of other acacias (Fig. 7c-e)–at the intermediate-rainfall site.

Herbivores of all size classes contributed to the suppression of growth in *A. mellifera* and *A. etbaica*. For *A. mellifera*, effects were dominated by mega- and mesoherbivores at low-rainfall sites, by dik-dik at intermediate-rainfall sites, and by megaherbivores at high-rainfall sites (Fig. 7c,d). Growth data for *A. etbaica* suggested the importance of dik-diks at low- and high-rainfall sites and megaherbivores at intermediate- and high-rainfall sites (Fig. 7e). Again, we suspect that for the most part, the observed differences in the relative influence of different LMH across sites are not caused by simple differences in relative abundance. Impala was the only browsing species whose dung density differed consistently across sites; this might partially explain the strong effects of mesoherbivore exclusion on *A. mellifera* growth at low-rainfall sites, but it does not explain the patterns (or lack thereof) observed for other woody species. We do note, however, that most of our permanently tagged trees are relatively large, with interquartile ranges for height varying from 1.62–2.30 m (*Croton dichogamus*) to 2.9–4.3 m (*Balanites aegyptiaca*). It seems likely that the effect of smaller browsers (dik-dik and impala) will be most pronounced for trees <2-m tall, with the impact of megaherbivores increasing beyond the 2-m threshold. We are therefore expanding the number of tagged trees in our dataset to allow a test of this prediction.

Across taxa, significant site*treatment interaction terms were uncommon. This may be explained in part by the conservative statistical approach adopted here (for the sake of providing a broad overview), which afforded limited power to detect such effects. We expected that increases in plant species’ abundance in exclosures (i.e., the suppressive effect of herbivory) would be most pronounced in low-rainfall sites [40], whereas any decreases in abundance within exclosures (e.g., resulting from overcompensation or intensified competition between plant species) would be strongest in high-rainfall sites [53]. This is what we observed for each of the understory plant species (*n = *4, or 7% of the total) that displayed significant site*treatment interactions. Likewise, increases in total understory and overstory plant densities within total-exclusion plots were most pronounced at low- and intermediate-rainfall sites. Small mammal densities, however, showed the opposite pattern (greatest increase in exclusion plots at the high-rainfall site), and the interactive effects on tree growth rates were variable. Thus, while context-dependent variability in the direct and indirect effects of LMH is clearly evident across the range of environmental conditions encompassed by UHURU, an integrated mechanistic explanation of this contingency remains a primary objective of our ongoing research.

## Supporting Information

Figure S1 **Size-selective large-herbivore barriers utilized in the UHURU experiment.** (A) Total exclusion; (B) intersection of total and mesoherbivore exclusion, the latter of which lacks a chain-link barrier at ground level; (C) megaherbivore exclusion, with wire at 2-m above ground level, allowing access to all herbivores smaller than elephant and giraffe; (D) open, which lacks fencing and has wooden posts at 10-m intervals to delineate plot boundaries. (Mohamud Mohamed has given written informed consent, as outlined in the PLOS consent form, to publication of his photograph.)
(TIF)

Figure S2 **Estimated biomass densities (left Y-axis) and metabolic loads (right Y-axis) for the three dominant large herbivores in each size class targeted by the UHURU experiment.** Biomass densities are taken from published estimates by Augustine (reference [55] in the main text). Metabolic load estimates are derived from biomass data using Nagy et al.’s allometric equations for field metabolic rates (reference [56] in the main text). Both estimates apply to the Mpala Conservancy as a whole, rather than to the experimental sites specifically.
(TIF)

Figure S3 Soil profiles at the three UHURU exclosure sites: (A) low-rainfall (north); (B) intermediate-rainfall (central); (C) high-rainfall (south). Details of soil profiles are provided in the main text and Text S1.
(TIF)

Figure S4 **Surface-soil composition.** (A) Relationship between percent clay and percent sand for each of the three experimental blocks at each site, showing outlying value in one block of the intermediate-rainfall site. (B) Non-metric multidimensional scaling plot showing compositional similarity of soils in each open and total-exclusion plot. This MDS analysis is based on 20 physical and chemical attributes, all of which were from 2012 samples except NO_3_ and NH_4_ (2010 data) and percent sand, silt, and clay (average of 2010 and 2012 data).
(TIF)

Figure S5 **Rank-abundance curves for understory plants at each site.** Curves were computed by summing the number of pin hits within each plot for each survey, averaging for each plot across the seven understory surveys conducted from 2008 to 2011, and then pooling the data for all plots within each site. Species codes for the six most abundant taxa at each site are as follows: *Cd* – *Cynodon dactylon*; *Cp* – *Cynodon plectostachyus*; *Ps* – *Pennisetum stramineum*; *Esp* – *Enteropogon* sp.; *Plsp* – *Plectranthus* sp. “small”; *Et* – *Eragrostis tenuifolia*; *Mk* – *Microchloa kunthii*; *Csp* – *Cymbopogon* sp.; *Bl* – *Brachiaria leersioides*; *T* – *Tragus* sp.
(TIF)

Figure S6 **Rank-abundance curves for overstory plants at each site.** Curves were computed by pooling data from all plots within each site for the 2012 woody-plant census. Species codes for the seven most abundant taxa at each site are as follows: *Ae* – *Acacia etbaica*; *Ab* – *Acacia brevispica*; *Eh* – *Euphorbia heterospina*; *Am* – *Acacia mellifera*; *Cd* – *Croton dichogamus*; *En – Euphorbia nyikae*; *G* – *Grewia* sp.; *T* – *Teclea* sp.; *R* – *Rhus* sp.; *C* – *Commiphora* sp.; *An* – *Acacia nilotica*; *L* – *Lycium* sp.
(TIF)

Table S1 **Mammal species known to occur at Mpala Research Centre, specifying those that have been observed (via direct observation or camera trapping) within at least one of the 36 UHURU plots between September 2008 and May 2012.**
(DOCX)

Table S2 **Raw surface-soil data for open and total-exclusion plots at each site, 2009–2012.** Means and standard deviations are derived from the three replicate plots of each treatment at each site. See main text for methodological details.
(DOCX)

Table S3 **Understory plant species for which no significant treatment effects were detected in linear mixed-model analyses with site, treatment, and their interaction as fixed effects, and with block as random effect (species showing significant treatment effects are listed in** **Table 2** **of the main text).** Effects of site were significant for twenty species, which are listed first. Tukey’s HSD post-hoc analyses were used to compare pin-hit frequencies at high-, intermediate-, and low-rainfall sites. Sites that do not share the same letter across these columns were significantly different (*P*≤0.05); the site with the highest frequency of a given species is always given the label “A”. Twenty-three species showed no significant effects of treatment, site, or their interaction. We did not analyze an additional 58 species for which we recorded fewer than 100 total pin hits (out of 123,480 total pins dropped in the course of seven surveys spanning 2008 to 2011). Species names preceded by superscript numerals were not initially not recognized as distinct and are therefore lumped in earlier surveys: ^1^ two *Aristida* spp., provisionally labeled “common” and “rare”, first distinguished in the fifth survey (October 2010); ^2^ two Barleria species, *B. acanthoides* and *B. ramulosa*, first distinguished in the seventh survey (October 2011); ^3^ *Hibiscus meyeri*, formerly labeled *Hibiscus* sp., first identified in the seventh survey; ^4^ two *Indigofera* species, provisionally labeled “big” and “small”, first distinguished in the fifth survey; ^5^ two Justicia species, provisionally labeled “white” and “pink”, first distinguished in the fifth survey; ^6^ two *Melhania* species, *M. velutina* and *M. ovata*, first distinguished in the seventh survey. Data for these species are thus drawn from one or from the average of three surveys.
(DOCX)

Text S1 Detailed descriptions of soil profiles at each site.
(DOCX)
