# Supplementary figures and images for: Piecewise Disassembly of a Large-Herbivore Community across a Rainfall Gradient: The UHURU Experiment
Source: PLoS One. 2013 Feb 6;8(2):e55192. doi: 10.1371/journal.pone.0055192 (PMC3566220; doi:10.1371/journal.pone.0055192)

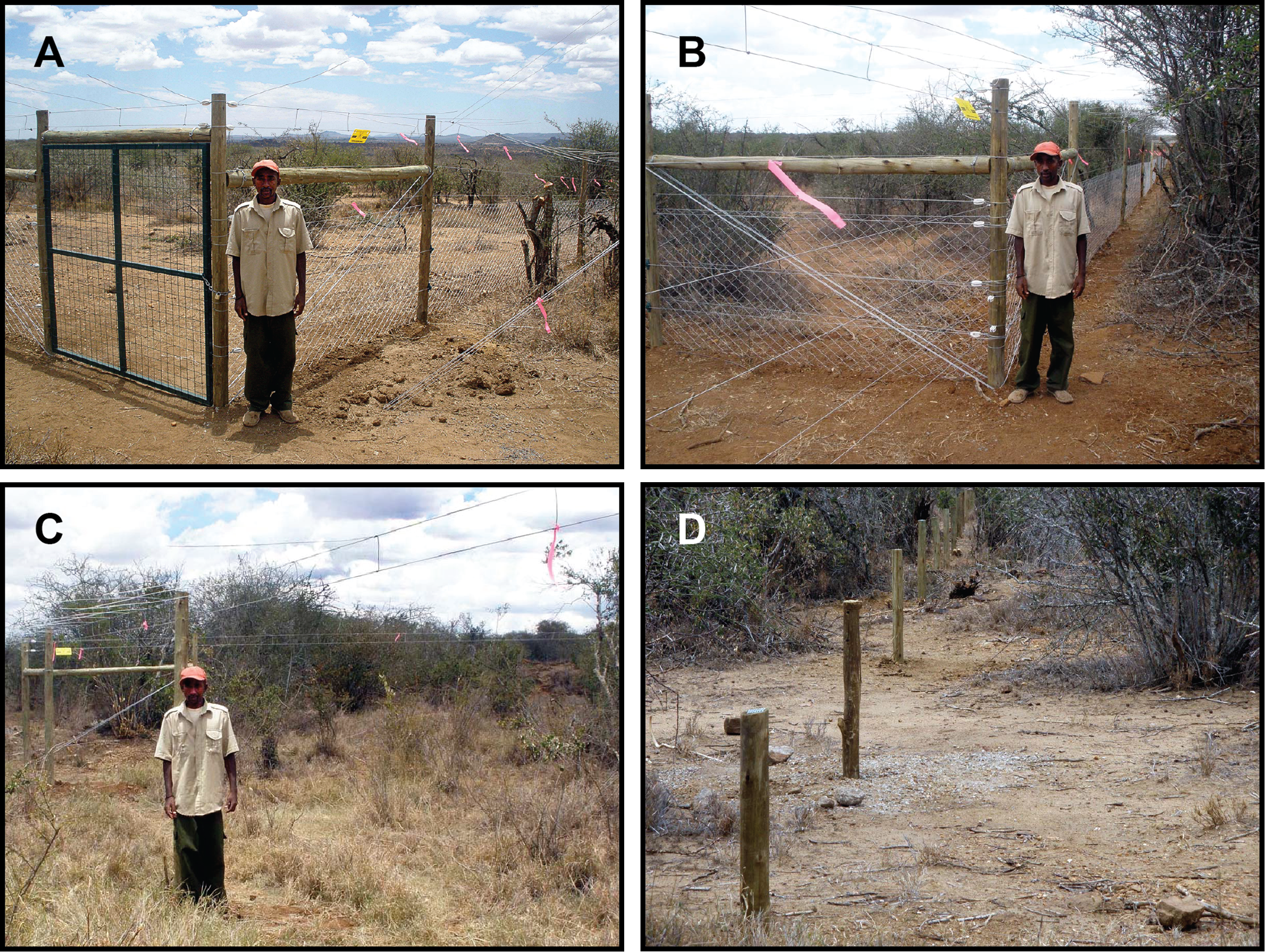

Supplement: Figure S1 — Size-selective large-herbivore barriers utilized in the UHURU experiment. (A) Total exclusion; (B) intersection of total and mesoherbivore exclusion, the latter of which lacks a chain-link barrier at ground level; (C) megaherbivore exclusion, with wire at 2-m above ground level, allowing access to all herbivores smaller than elephant and giraffe; (D) open, which lacks fencing and has wooden posts at 10-m intervals to delineate plot boundaries. (Mohamud Mohamed has given written informed consent, as outlined in the PLOS consent form, to publication of his photograph.) (TIF) [file pone.0055192.s001.tif]

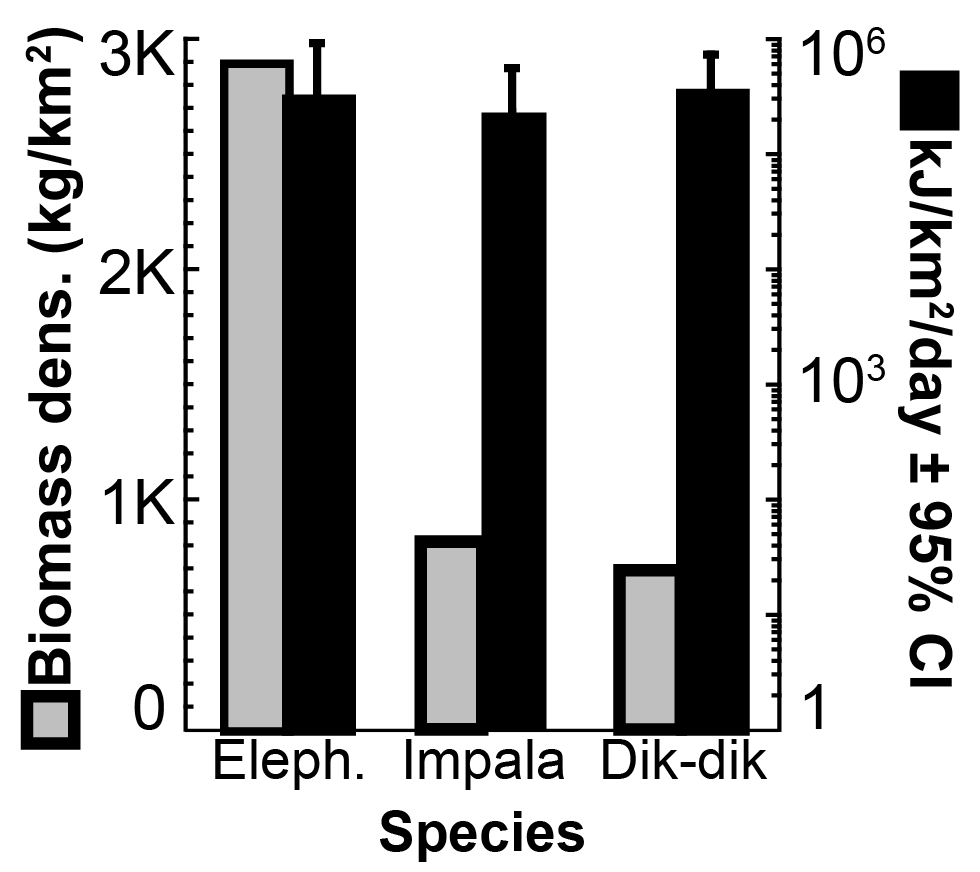

Supplement: Figure S2 — Estimated biomass densities (left Y-axis) and metabolic loads (right Y-axis) for the three dominant large herbivores in each size class targeted by the UHURU experiment. Biomass densities are taken from published estimates by Augustine (reference [55] in the main text). Metabolic load estimates are derived from biomass data using Nagy et al.’s allometric equations for field metabolic rates (reference [56] in the main text). Both estimates apply to the Mpala Conservancy as a whole, rather than to the experimental sites specifically. (TIF) [file pone.0055192.s002.tif]

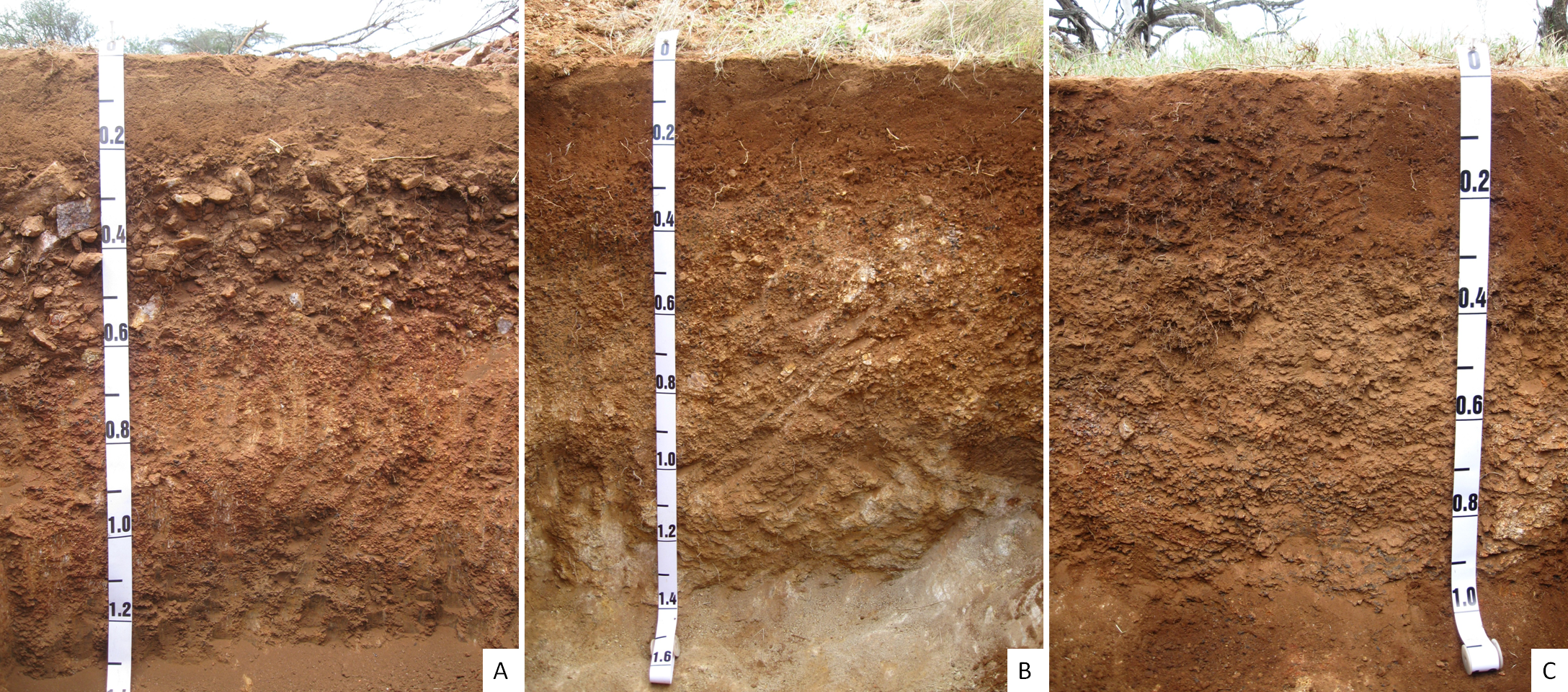

Supplement: Figure S3 — Soil profiles at the three UHURU exclosure sites: (A) low-rainfall (north); (B) intermediate-rainfall (central); (C) high-rainfall (south). Details of soil profiles are provided in the main text and Text S1. (TIF) [file pone.0055192.s003.tif]

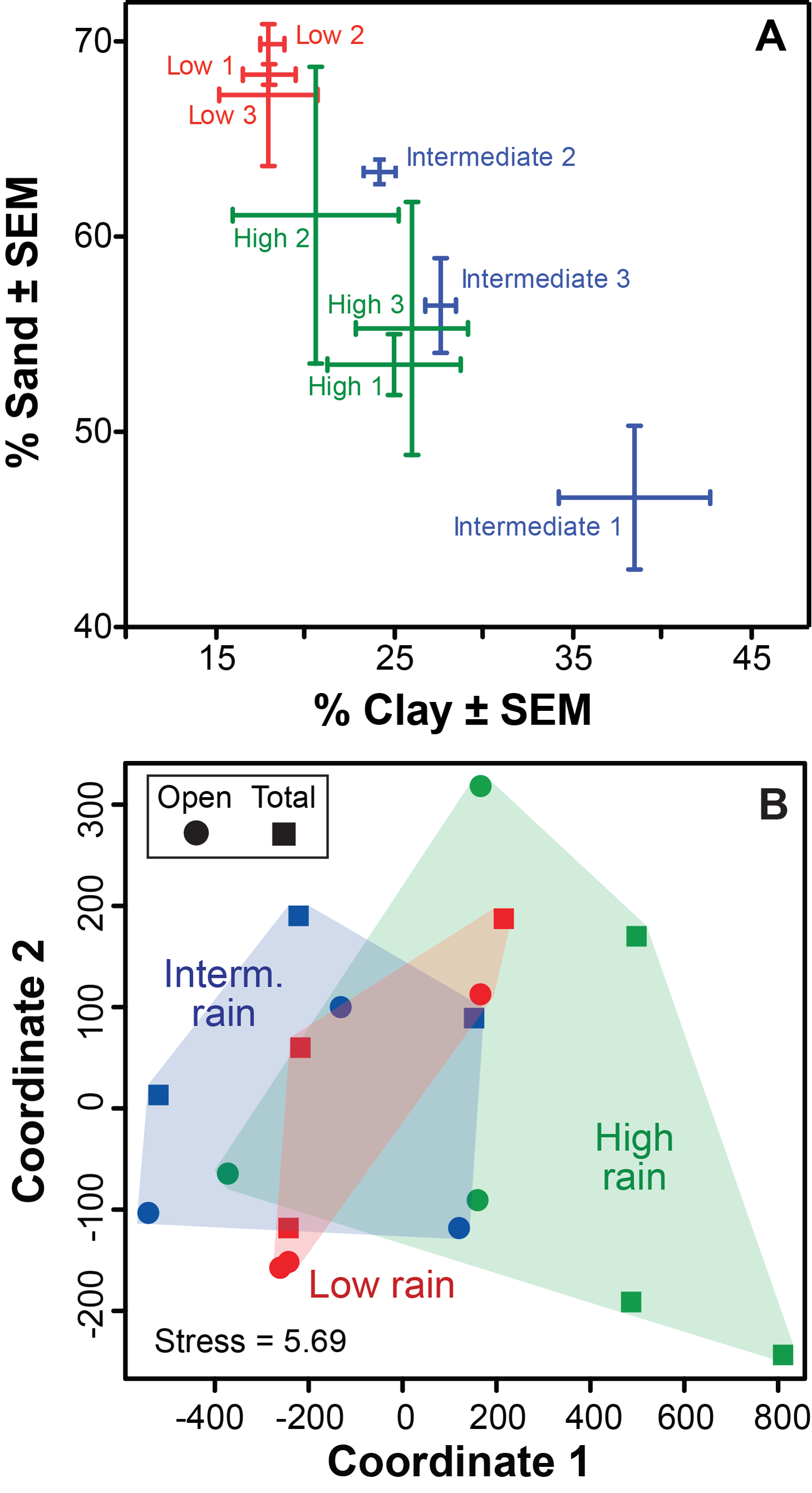

Supplement: Figure S4 — Surface-soil composition. (A) Relationship between percent clay and percent sand for each of the three experimental blocks at each site, showing outlying value in one block of the intermediate-rainfall site. (B) Non-metric multidimensional scaling plot showing compositional similarity of soils in each open and total-exclusion plot. This MDS analysis is based on 20 physical and chemical attributes, all of which were from 2012 samples except NO3 and NH4 (2010 data) and percent sand, silt, and clay (average of 2010 and 2012 data). (TIF) [file pone.0055192.s004.tif]

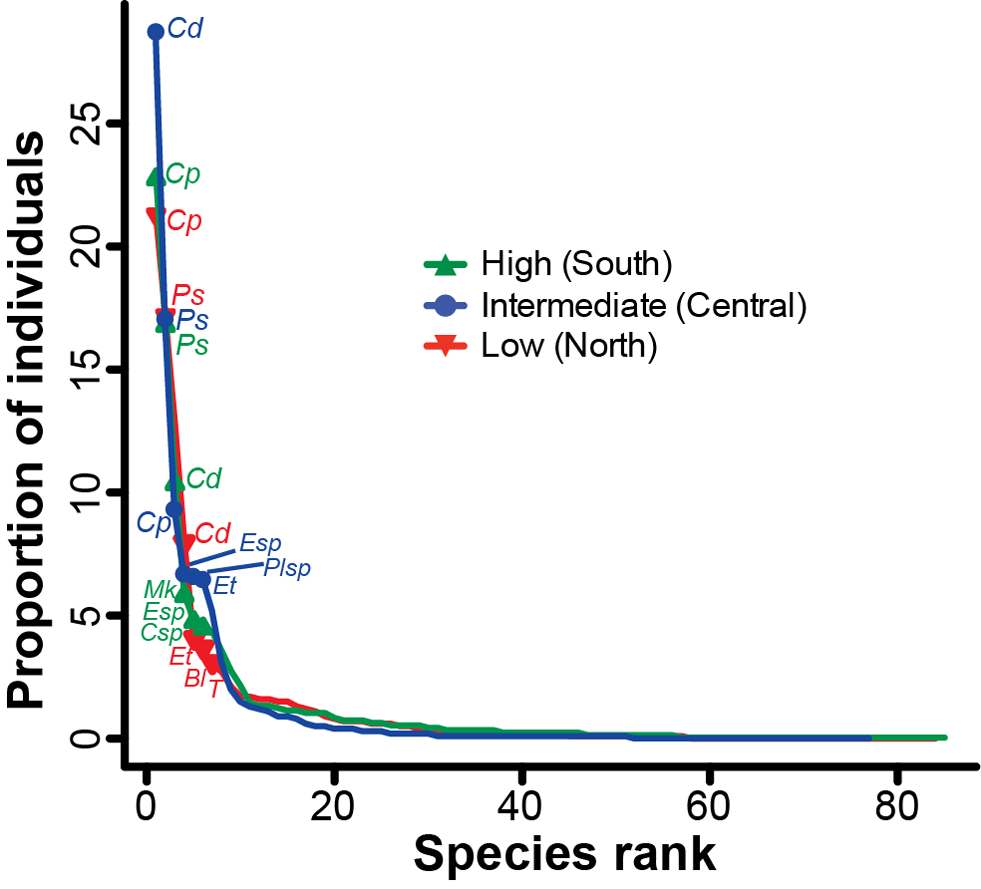

Supplement: Figure S5 — Rank-abundance curves for understory plants at each site. Curves were computed by summing the number of pin hits within each plot for each survey, averaging for each plot across the seven understory surveys conducted from 2008 to 2011, and then pooling the data for all plots within each site. Species codes for the six most abundant taxa at each site are as follows: Cd – Cynodon dactylon; Cp – Cynodon plectostachyus; Ps – Pennisetum stramineum; Esp – Enteropogon sp.; Plsp – Plectranthus sp. “small”; Et – Eragrostis tenuifolia; Mk – Microchloa kunthii; Csp – Cymbopogon sp.; Bl – Brachiaria leersioides; T – Tragus sp. (TIF) [file pone.0055192.s005.tif]

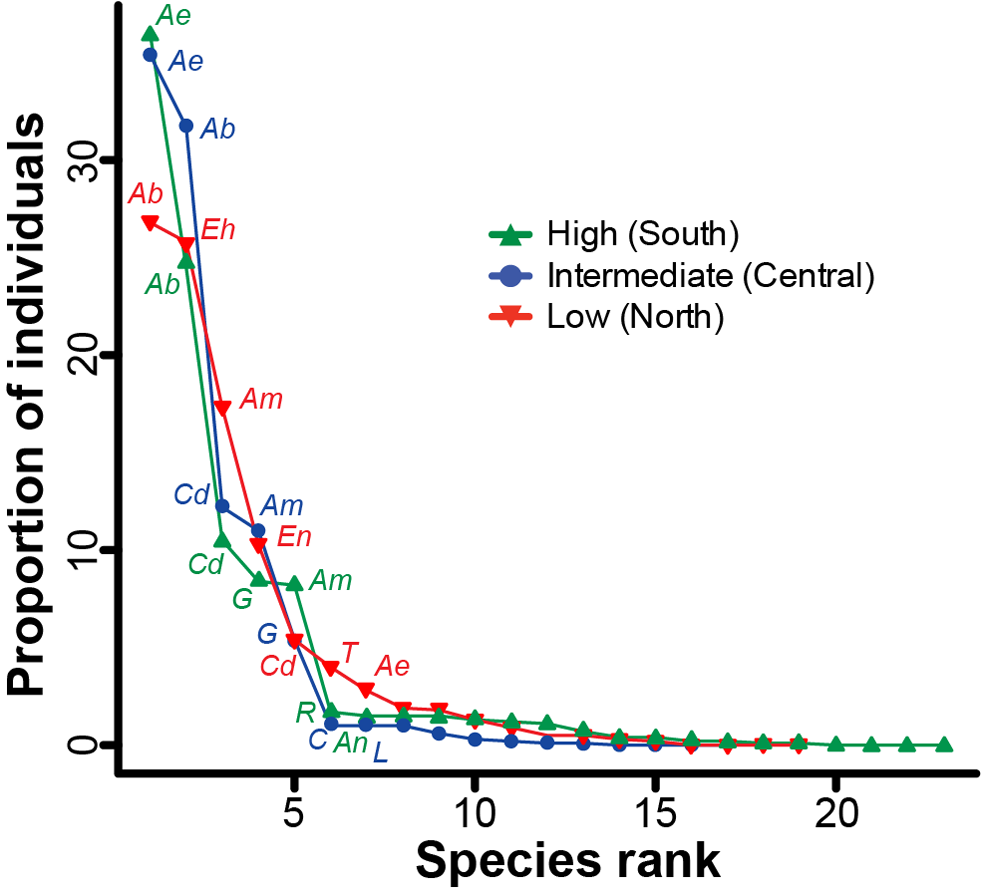

Supplement: Figure S6 — Rank-abundance curves for overstory plants at each site. Curves were computed by pooling data from all plots within each site for the 2012 woody-plant census. Species codes for the seven most abundant taxa at each site are as follows: Ae – Acacia etbaica; Ab – Acacia brevispica; Eh – Euphorbia heterospina; Am – Acacia mellifera; Cd – Croton dichogamus; En – Euphorbia nyikae; G – Grewia sp.; T – Teclea sp.; R – Rhus sp.; C – Commiphora sp.; An – Acacia nilotica; L – Lycium sp. (TIF) [file pone.0055192.s006.tif]
