# Supplementary material for: Piecewise Disassembly of a Large-Herbivore Community across a Rainfall Gradient: The UHURU Experiment
Source: PLoS One. 2013 Feb 6;8(2):e55192. doi: 10.1371/journal.pone.0055192 (PMC3566220; doi:10.1371/journal.pone.0055192)
Supplement: Table S1 — Mammal species known to occur at Mpala Research Centre, specifying those that have been observed (via direct observation or camera trapping) within at least one of the 36 UHURU plots between September 2008 and May 2012. (DOCX) [file pone.0055192.s007.docx]

**Table S1**

Mammal species known to occur at Mpala Research Centre, specifying those that have been observed (via direct observation or camera trapping) within at least one of the 36 UHURU plots between September 2008 and May 2012.

| Species | Recorded in UHURU plots? |
| --- | --- |
| Large herbivores (≥ 5 kg) |  |
| Elephant (*Loxodonta africana*) | **Yes** |
| Hippopotamus (*Hippopotamus amphibius*) |  |
| Reticulated giraffe (*Giraffa camelopardalis*) | **Yes** |
| Buffalo (*Syncerus caffer*) | **Yes** |
| Grevy's zebra (*Equus grevyi*) | **Yes** |
| Plains zebra (*Equus quagga*) | **Yes** |
| Bushpig (*Potamochoerus larvatus*) |  |
| Warthog (*Phacochoerus africanus*) | **Yes** |
| Eland (*Taurotragus oryx*) | **Yes** |
| Greater kudu (*Tragelaphus strepsiceros*) |  |
| Oryx (*Oryx beisa*) | **Yes** |
| Waterbuck (*Kobus defassa*) | **Yes** |
| Hartebeest (*Alcelaphus buselaphus*) |  |
| Impala (*Aepyceros melampus*) | **Yes** |
| Gerenuk (*Litocranius walleri*) | **Yes** |
| Grant's gazelle (*Nanger granti*) |  |
| Bushbuck (*Tragelaphus sylvaticus*) |  |
| Thomson's gazelle (*Eudorcas thomsoni*) |  |
| Bush duiker (*Sylvicapra grimmia*) |  |
| Steinbuck (*Raphicerus campestris*) |  |
| Klipspringer (*Oreotragus aureus*) | **Yes** |
| Dik-dik (*Madoqua cavendishi*) | **Yes** |
| Cattle (*Bos primigenius*) | **Yes** |
| Donkey (*Equus africanus*) | **Yes** |
| Camel (*Camelus dromedaries*) |  |
| SMALL MAMMALS | |
| Hares *(Lepus* *capensis* and *L. saxatilis*) | **Yes** |
| Porcupine (*Hystrix cristata*) | **Yes** |
| Rufous elephant shrew (*Elephantulus rufescens*) | **Yes** |
| Hedgehog (*Ateletrix albiventris*) | **Yes** |
| Shrews (*Crocidura* sp.) | **Yes** |
| Striped ground squirrel (*Xerus erythropus*) | **Yes** |
| Ochre bush squirrel (*Paraxerus ochraceus*) | **Yes** |
| Kemp's spiny mouse (*Acomys kempi*) | **Yes** |
| Percival's spiny mouse (*Acomys percivali*) | **Yes** |
| Hinde's rock rat (*Aethomys hindei*) | **Yes** |
| Nairobi grass rat (*Arvicanthis nairobae*) | **Yes** |
| African grass rat (*Arvicanthis niloticus*) | **Yes** |
| Climbing mouse (*Dendromus* sp.) | **Yes** |
| Fringe-tailed gerbil (*Gerbilliscus robustus*) | **Yes** |
| Harrington's tateril (*Taterillus harringtoni*) | **Yes** |
| Woodland thicket rat (*Grammomys dolichurus*) | **Yes** |
| Natal multi-mammate rat (*Mastomys natalensis*) | **Yes** |
| Northern pouched mouse (*Saccostomus mearnsi*) | **Yes** |
| Pygmy mice (*Mus* spp., including *M. minitoides* and *M. musculoides*) | **Yes** |
| CARNIVORES | |
| Lion (*Panthera leo*) | **Yes** |
| Leopard (*Panthera pardus*) | **Yes** |
| Cheetah (*Acinonyx jubatus*) |  |
| Serval (*Leptailurus serval*) |  |
| Caracal (*Caracal caracal*) | **Yes** |
| Wild cat (*Felis lybica*) | **Yes** |
| Spotted hyena (*Crocuta crocuta*) | **Yes** |
| Striped hyena *(Hyaena* *hyaena*) | **Yes** |
| Aardwolf (*Proteles cristatus*) | **Yes** |
| Wild dog *(Lycaon* *pictus)* | **Yes** |
| Black-backed jackal (*Canis mesomelas*) | **Yes** |
| Bat-eared fox (*Otocyon megalotis*) |  |
| Honey badger (Mellivora capensis) | **Yes** |
| Common genet (*Genetta genetta*) | **Yes** |
| White-tailed mongoose (*Ichneumia albicauda*) | **Yes** |
| Slender mongoose (*Galerella* *sanguinea*) | **Yes** |
| Dwarf mongoose (*Helogale parvula*) | **Yes** |
| Zorilla (*Ictonyx striatus*) |  |
| OTHER SPECIES | |
| Aardvark (*Orycteropus afer*) | **Yes** |
| Baboon (*Papio anubis*) | **Yes** |
| Vervet monkey (*Chlorocebus pygerythrus*) | **Yes** |
| Galago (*Galago senegalensis*) |  |
