# Supplementary material for: Piecewise Disassembly of a Large-Herbivore Community across a Rainfall Gradient: The UHURU Experiment
Source: PLoS One. 2013 Feb 6;8(2):e55192. doi: 10.1371/journal.pone.0055192 (PMC3566220; doi:10.1371/journal.pone.0055192)
Supplement: Table S2 — Raw surface-soil data for open and total-exclusion plots at each site, 2009–2012. Means and standard deviations are derived from the three replicate plots of each treatment at each site. See main text for methodological details. (DOCX) [file pone.0055192.s008.docx]

**Table S2**

Raw surface-soil data for open and total-exclusion plots at each site, 2009-2012. Means and standard deviations are derived from the three replicate plots of each treatment at each site. See main text for methodological details.

|  | Low (North) | | | | Intermediate (Central) | | | | High (South) | | | |
| --- | --- | --- | --- | --- | --- | --- | --- | --- | --- | --- | --- | --- |
|  | **Open** | | **Total** | | **Open** | | **Total** | | **Open** | | **Total** | |
|  | **Mean** | **SD** | **Mean** | **SD** | **Mean** | **SD** | **Mean** | **SD** | **Mean** | **SD** | **Mean** | **SD** |
| 2009 samples |  |  |  |  |  |  |  |  |  |  |  |  |
| pH | 5.87 | 0.06 | 6.37 | 0.29 | 5.37 | 0.35 | 5.60 | 0.44 | 6.17 | 0.21 | 6.13 | 0.47 |
| Exch. K (cmol_c_/kg) | 0.66 | 0.06 | 0.59 | 0.08 | 0.79 | 0.13 | 0.70 | 0.12 | 0.84 | 0.04 | 1.05 | 0.17 |
| Exch. Ca (cmol_c_/kg) | 4.63 | 1.55 | 5.27 | 1.12 | 5.47 | 2.20 | 5.03 | 1.17 | 6.03 | 1.76 | 5.80 | 2.16 |
| Exch. Mg (cmol_c_/kg) | 2.03 | 0.58 | 2.33 | 0.49 | 3.00 | 1.31 | 2.37 | 1.00 | 3.13 | 0.81 | 2.37 | 1.00 |
| P (ppm) | 7.5 | 1.9 | 5.0 | 1.4 | 4.3 | 1.7 | 4.7 | 1.1 | 4.1 | 0.6 | 12.7 | 10.1 |
| % Total C | 0.88 | 0.10 | 1.02 | 0.08 | 0.90 | 0.19 | 0.79 | 0.09 | 1.07 | 0.28 | 0.99 | 0.24 |
| % Total N | 0.10 | 0.02 | 0.11 | 0.01 | 0.11 | 0.02 | 0.10 | 0.01 | 0.12 | 0.03 | 0.11 | 0.02 |
| 2010 samples |  |  |  |  |  |  |  |  |  |  |  |  |
| % Clay | 15.4 | 0.3 | 14.6 | 0.4 | 19.3 | 3.8 | 19.4 | 3.0 | 16.0 | 1.3 | 18.7 | 1.5 |
| % Sand | 80.0 | 0.9 | 79.5 | 1.5 | 76.0 | 4.8 | 75.7 | 4.6 | 78.2 | 2.1 | 76.5 | 1.7 |
| % Silt | 4.6 | 0.7 | 5.8 | 1.2 | 4.7 | 1.2 | 5.0 | 1.6 | 5.8 | 1.3 | 4.8 | 1.4 |
| NH4 (ppm) | 13.1 | 3.0 | 9.0 | 2.2 | 12.9 | 4.5 | 10.0 | 0.6 | 13.2 | 4.7 | 12.5 | 0.2 |
| NO3 (ppm) | 5.9 | 0.5 | 13.6 | 13.0 | 15.0 | 3.7 | 15.9 | 7.5 | 16.1 | 10.6 | 9.3 | 4.2 |
| 2012 samples |  |  |  |  |  |  |  |  |  |  |  |  |
| % Clay | 17.9 | 2.3 | 18.3 | 2.2 | 31.5 | 10.1 | 28.7 | 4.9 | 22.6 | 6.4 | 25.3 | 3.9 |
| % Sand | 69.5 | 1.6 | 67.4 | 3.4 | 53.2 | 9.9 | 57.7 | 6.9 | 60.8 | 8.5 | 52.4 | 3.2 |
| % Silt | 12.6 | 1.3 | 14.3 | 1.6 | 15.2 | 1.9 | 13.6 | 2.3 | 16.7 | 2.3 | 22.3 | 1.3 |
| pH | 5.83 | 0.42 | 5.97 | 0.32 | 5.00 | 0.52 | 5.27 | 0.42 | 6.33 | 0.21 | 6.13 | 0.06 |
| Infiltration Rate (mm/min) | 1.86 | 0.75 | 1.93 | 1.81 | 1.31 | 0.74 | 1.86 | 0.51 | 2.00 | 1.36 | 1.95 | 0.55 |
| Al (mg/kg) | 611.0 | 18.2 | 622.3 | 32.3 | 848.7 | 72.0 | 840.0 | 58.8 | 742.0 | 94.9 | 717.7 | 101.8 |
| Avail. P (Bray 1, ppm) | 9.67 | 0.58 | 10.67 | 0.58 | 5.33 | 2.31 | 6.33 | 2.52 | 4.33 | 0.58 | 14.33 | 15.37 |
| B (mg/kg) | 0.46 | 0.14 | 0.49 | 0.06 | 0.34 | 0.10 | 0.34 | 0.15 | 0.37 | 0.08 | 0.48 | 0.08 |
| Ca (mg/kg) | 1129 | 268 | 1192 | 269 | 1057 | 304 | 1093 | 322 | 1254 | 328 | 1782 | 137 |
| Cu (mg/kg) | 1.33 | 0.14 | 1.37 | 0.15 | 1.06 | 0.31 | 1.12 | 0.21 | 1.18 | 0.17 | 1.42 | 0.08 |
| Fe (mg/kg) | 58.67 | 2.52 | 57.67 | 0.58 | 59.33 | 8.33 | 68.00 | 4.58 | 75.67 | 8.50 | 70.33 | 13.80 |
| K (mg/kg) | 506.7 | 69.0 | 412.3 | 86.8 | 479.7 | 156.9 | 339.0 | 112.6 | 432.0 | 201.6 | 763.3 | 245.4 |
| Mg (mg/kg) | 296.0 | 67.9 | 299.7 | 107.6 | 365.0 | 113.5 | 320.0 | 52.2 | 328.7 | 81.7 | 401.7 | 50.8 |
| Mn (mg/kg) | 129.0 | 22.6 | 138.7 | 48.9 | 110.0 | 4.6 | 100.7 | 29.7 | 133.0 | 18.2 | 149.0 | 27.6 |
| Na (mg/kg) | 29.7 | 8.6 | 26.0 | 6.2 | 48.0 | 13.5 | 33.3 | 4.6 | 27.3 | 4.5 | 36.7 | 0.6 |
| % Organic Matter | 1.60 | 0.28 | 1.98 | 0.19 | 2.43 | 0.62 | 2.14 | 0.32 | 2.24 | 0.41 | 2.46 | 0.36 |
| S (ppm) | 19.3 | 3.2 | 18.7 | 10.0 | 38.3 | 17.6 | 24.0 | 8.0 | 9.3 | 2.5 | 21.0 | 10.0 |
| Zn (mg/kg) | 1.32 | 0.27 | 1.39 | 0.25 | 0.71 | 0.29 | 0.76 | 0.29 | 0.97 | 0.11 | 1.63 | 1.51 |
| Total Exch. Cap. (cmol_c_/kg) | 13.1 | 0.7 | 12.9 | 4.6 | 20.5 | 7.8 | 16.1 | 1.0 | 12.0 | 2.3 | 17.6 | 1.7 |
