# Supplementary material for: Piecewise Disassembly of a Large-Herbivore Community across a Rainfall Gradient: The UHURU Experiment
Source: PLoS One. 2013 Feb 6;8(2):e55192. doi: 10.1371/journal.pone.0055192 (PMC3566220; doi:10.1371/journal.pone.0055192)
Supplement: Table S3 — Understory plant species for which no significant treatment effects were detected in linear mixed-model analyses with site, treatment, and their interaction as fixed effects, and with block as random effect (species showing significant treatment effects are listed in Table 2 of the main text). Effects of site were significant for twenty species, which are listed first. Tukey’s HSD post-hoc analyses were used to compare pin-hit frequencies at high-, intermediate-, and low-rainfall sites. Sites that do not share the same letter across these columns were significantly different (P≤0.05); the site with the highest frequency of a given species is always given the label “A”. Twenty-three species showed no significant effects of treatment, site, or their interaction. We did not analyze an additional 58 species for which we recorded fewer than 100 total pin hits (out of 123,480 total pins dropped in the course of seven surveys spanning 2008 to 2011). Species names preceded by superscript numerals were not initially not recognized as distinct and are therefore lumped in earlier surveys: 1 two Aristida spp., provisionally labeled “common” and “rare”, first distinguished in the fifth survey (October 2010); 2 two Barleria species, B. acanthoides and B. ramulosa, first distinguished in the seventh survey (October 2011); 3 Hibiscus meyeri, formerly labeled Hibiscus sp., first identified in the seventh survey; 4 two Indigofera species, provisionally labeled “big” and “small”, first distinguished in the fifth survey; 5 two Justicia species, provisionally labeled “white” and “pink”, first distinguished in the fifth survey; 6 two Melhania species, M. velutina and M. ovata, first distinguished in the seventh survey. Data for these species are thus drawn from one or from the average of three surveys. (DOCX) [file pone.0055192.s009.docx]

**Table S3**

Understory plant species for which no significant treatment effects were detected in linear mixed-model analyses with site, treatment, and their interaction as fixed effects, and with block as random effect (species showing significant treatment effects are listed in Table 2 of the main text). Effects of site were significant for twenty species, which are listed first. Tukey’s HSD post-hoc analyses were used to compare pin-hit frequencies at high-, intermediate-, and low-rainfall sites. Sites that do not share the same letter across these columns were significantly different (*P* ≤ 0.05); the site with the highest frequency of a given species is always given the label “A”. Twenty-three species showed no significant effects of treatment, site, or their interaction. We did not analyze an additional 58 species for which we recorded fewer than 100 total pin hits (out of 123,480 total pins dropped in the course of seven surveys spanning 2008 to 2011). Species names preceded by superscript numerals were not initially not recognized as distinct and are therefore lumped in earlier surveys: ^1^ two *Aristida* spp., provisionally labeled “common” and “rare”, first distinguished in the fifth survey (October 2010); ^2^ two Barleria species, *B. acanthoides* and *B. ramulosa*, first distinguished in the seventh survey (October 2011); ^3^ *Hibiscus meyeri*, formerly labeled *Hibiscus* sp., first identified in the seventh survey; ^4^ two *Indigofera* species, provisionally labeled “big” and “small”, first distinguished in the fifth survey; ^5^ two Justicia species, provisionally labeled “white” and “pink”, first distinguished in the fifth survey; ^6^ two *Melhania* species, *M. velutina* and *M. ovata*, first distinguished in the seventh survey. Data for these species are thus drawn from one or from the average of three surveys.

| Species | Total hits | Rank abund. | Treat-ment *P* | Site  *P* | High | | Int. | | Low | | Treatment x Site | |  |
| --- | --- | --- | --- | --- | --- | --- | --- | --- | --- | --- | --- | --- | --- |
| *Microchloa kunthii* | 4356 | 6 | 0.68 | **0.003** | | A | | B | | B | | 0.83 | |
| *Themeda triandra* | 2650 | 7 | 0.81 | **0.003** | | A | | B | | B | | 0.73 | |
| *Cymbopogon* sp. | 2523 | 8 | 0.27 | **0.02** | | A | | B | | B | | 0.16 | |
| *Tragus* sp. | 1727 | 11 | 0.29 | **0.001** | | B | | B | | A | | 0.57 | |
| *Portulaca quadrifida* | 898 | 19 | 0.11 | **0.019** | | A | | B | | A,B | | 0.37 | |
| *Gutenbergia* sp. | 877 | 20 | 0.32 | **0.02** | | A,B | | B | | A | | 0.38 | |
| *Eragrostis superba* | 770 | 22 | 0.10 | **0.008** | | A | | B | | B | | 0.10 | |
| *Eragrostis racemosa* | 525 | 25 | 0.54 | **0.01** | | B | | B | | A | | 0.75 | |
| *Digitaria milanjiana* | 512 | 28 | 0.69 | **0.001** | | A | | B | | B | | 0.94 | |
| *Hibiscus calyphyllus* | 369 | 34 | 0.12 | **0.05** | | A | | A | | A | | 0.27 | |
| *Chloris gayana* | 341 | 35 | 0.62 | **0.003** | | B | | B | | A | | 0.61 | |
| *Ocimum* sp. | 337 | 37 | 0.40 | **0.04** | | A | | B | | A,B | | 0.77 | |
| *Harpachne schimperi* | 310 | 40 | 0.08 | **0.02** | | A | | B | | B | | 0.07 | |
| ^4^*Indigofera* sp. "big" | 273 | 43 | 0.69 | **0.004** | | B | | B | | A | | 0.83 | |
| *Phyllanthus* sp. | 248 | 44 | 0.83 | **0.0007** | | B | | B | | A | | 0.71 | |
| *Sida* sp. | 237 | 45 | 0.26 | **0.01** | B | | B | | A | | **0.05** | |  |
| ^1^*Aristida* sp. "rare" | 197 | 48 | 0.54 | **0.02** | A,B | | A | | B | | 0.57 | |  |
| *Pentanisia* sp. | 132 | 52 | 0.50 | **0.04** | A | | B | | A,B | | 0.82 | |  |
| ^2^*Barleria ramulosa* | 131 | 53 | 0.58 | **0.003** | B | | B | | A | | 0.63 | |  |
| *Osteospermum* sp. | 119 | 55 | 0.12 | **0.01** | A | | B | | A,B | | 0.15 | |  |
| *Eragrostis tenuifolia* | 6732 | 4 | 0.44 | 0.16 | - | | - | | - | | 0.55 | |  |
| *Enteropogon* sp. | 4621 | 5 | 0.64 | 0.24 | - | | - | | - | | 0.75 | |  |
| ^1^*Aristida* sp. "common" | 1671 | 12 | 0.98 | 0.83 | - | | - | | - | | 0.35 | |  |
| *Barleria spinisepala* | 1343 | 14 | 0.59 | 0.52 | - | | - | | - | | 0.37 | |  |
| *Cyathula* sp. | 1074 | 16 | 0.25 | 0.22 | - | | - | | - | | 0.96 | |  |
| *Pennisetum mezianum* | 1053 | 17 | 0.26 | 0.61 | - | | - | | - | | 0.62 | |  |
| *Chloris roxburghiana* | 924 | 18 | 0.33 | 0.17 | - | | - | | - | | 0.52 | |  |
| *Hibiscus* sp. "2" | 864 | 21 | 0.14 | 0.07 | - | | - | | - | | 0.22 | |  |
| *Pollichia* sp. | 690 | 24 | 0.17 | 0.09 | - | | - | | - | | 0.46 | |  |
| *Eragrostis papposa* | 522 | 26 | 0.69 | 0.30 | - | | - | | - | | 0.76 | |  |
| *Dactyloctenium* sp. | 461 | 29 | 0.13 | 0.26 | - | | - | | - | | 0.67 | |  |
| ^5^*Justicia* sp. "white" | 428 | 31 | 0.40 | 0.35 | - | | - | | - | | 0.27 | |  |
| *Bothriochloa insculpta* | 400 | 32 | 0.53 | 0.08 | - | | - | | - | | 0.42 | |  |
| *Dinebra* sp. | 337 | 36 | 0.51 | 0.10 | - | | - | | - | | 0.58 | |  |
| *Panicum maximum* | 322 | 39 | 0.13 | 0.14 | - | | - | | - | | 0.46 | |  |
| *Sporobolus ioclados* | 303 | 41 | 0.99 | 0.19 | - | | - | | - | | 0.12 | |  |
| *Barleria* sp. "2" | 286 | 42 | 0.30 | 0.47 | - | | - | | - | | 0.82 | |  |
| *Abutilon* sp. | 230 | 46 | 0.39 | 0.19 | - | | - | | - | | 0.55 | |  |
| *Cassia* sp. | 191 | 49 | 0.14 | 0.81 | - | | - | | - | | 0.80 | |  |
| *Pavonia* sp. | 167 | 50 | 0.46 | 0.11 | - | | - | | - | | 0.95 | |  |
| *Achyranthes aspera* | 140 | 51 | 0.58 | 0.12 | - | | - | | - | | 0.83 | |  |
| *Lippia* sp. | 121 | 54 | 0.32 | 0.38 | - | | - | | - | | 0.64 | |  |
| *Sporobolus africanus* | 101 | 56 | 0.38 | 0.08 | - | | - | | - | | 0.40 | |  |
| *Eragrostis* sp. | 99 | 57 |  |  |  | |  | |  | |  | |  |
| *Tribulus* sp. | 98 | 58 |  |  |  | |  | |  | |  | |  |
| *Hibiscus aponeurus* | 80 | 59 |  |  |  | |  | |  | |  | |  |
| *Plectranthus* sp. "big" | 65 | 60 |  |  |  | |  | |  | |  | |  |
| ^2^*Barleria acanthoides* | 62 | 61 |  |  |  | |  | |  | |  | |  |
| ^5^*Justicia* sp. "pink" | 59 | 62 |  |  |  | |  | |  | |  | |  |
| ^3^*Hibiscus meyeri* | 56 | 63 |  |  |  | |  | |  | |  | |  |
| *Ipomoea* sp. "2" | 56 | 64 |  |  |  | |  | |  | |  | |  |
| *Euphorbia heterospina*  *Euphorbia heterospina* | 51 | 65 |  |  |  | |  | |  | |  | |  |
| Lily sp. | 46 | 66 |  |  |  | |  | |  | |  | |  |
| *Oxygonum* sp. | 45 | 67 |  |  |  | |  | |  | |  | |  |
| *Leucas* sp. | 40 | 68 |  |  |  | |  | |  | |  | |  |
| *Setaria* sp. | 38 | 69 |  |  |  | |  | |  | |  | |  |
| *Polygala* sp. | 30 | 70 |  |  |  | |  | |  | |  | |  |
| *Cucumis* sp. | 29 | 71 |  |  |  | |  | |  | |  | |  |
| *Crabbea* sp. | 27 | 72 |  |  |  | |  | |  | |  | |  |
| *Hibiscus flavifolius* | 27 | 73 |  |  |  | |  | |  | |  | |  |
| ^6^*Melhania ovata* sp. | 21 | 74 |  |  |  | |  | |  | |  | |  |
| *Tagetes* sp. | 20 | 75 |  |  |  | |  | |  | |  | |  |
| *Phyllanthus* sp. "was cassia" | 19 | 76 |  |  |  | |  | |  | |  | |  |
| *Sarcostemma* sp. | 19 | 77 |  |  |  | |  | |  | |  | |  |
| *Emilia* sp. | 17 | 78 |  |  |  | |  | |  | |  | |  |
| *Kalanchoe* sp. | 16 | 79 |  |  |  | |  | |  | |  | |  |
| *Sansevieria perrotii* (syn. *S. robusta*) | 16 | 80 |  |  |  | |  | |  | |  | |  |
| unknown sp. "4" | 16 | 81 |  |  |  | |  | |  | |  | |  |
| *Euphorbia nyikae* | 15 | 82 |  |  |  | |  | |  | |  | |  |
| unknown sp. "30" | 15 | 83 |  |  |  | |  | |  | |  | |  |
| *Amaranthus* sp. | 13 | 84 |  |  |  | |  | |  | |  | |  |
| *Justicia odora* | 12 | 85 |  |  |  | |  | |  | |  | |  |
| *Chenopodium* sp. | 11 | 86 |  |  |  | |  | |  | |  | |  |
| *Priva* sp. | 9 | 87 |  |  |  | |  | |  | |  | |  |
| unknown sp. "27" | 9 | 88 |  |  |  | |  | |  | |  | |  |
| ^6^*Melhania velutina* | 8 | 89 |  |  |  | |  | |  | |  | |  |
| *Barleria* sp. "3" | 8 | 90 |  |  |  | |  | |  | |  | |  |
| *Felicia muricata* | 7 | 91 |  |  |  | |  | |  | |  | |  |
| *Sporobolus* sp. | 7 | 92 |  |  |  | |  | |  | |  | |  |
| Fern sp. | 6 | 93 |  |  |  | |  | |  | |  | |  |
| *Euphorbia* sp. "rare" | 5 | 94 |  |  |  | |  | |  | |  | |  |
| *Portulaca* sp. | 5 | 95 |  |  |  | |  | |  | |  | |  |
| *Ipomoea kituiensis* | 4 | 96 |  |  |  | |  | |  | |  | |  |
| *Ipomoea* sp. "3" | 3 | 97 |  |  |  | |  | |  | |  | |  |
| *Kleinia odora* | 3 | 98 |  |  |  | |  | |  | |  | |  |
| unknown sp. "*2"* | 2 | 99 |  |  |  | |  | |  | |  | |  |
| unknown sp. "5" | 2 | 100 |  |  |  | |  | |  | |  | |  |
| unknown sp. "31" | 2 | 101 |  |  |  | |  | |  | |  | |  |
| *Caralluma* sp. | 1 | 102 |  |  |  | |  | |  | |  | |  |
| *Craterostigma* sp. | 1 | 103 |  |  |  | |  | |  | |  | |  |
| *Monechma* cf. *debile* | 1 | 104 |  |  |  | |  | |  | |  | |  |
| *Melinis* (syn. *Rhynchelytrum*) *repens* | 1 | 105 |  |  |  | |  | |  | |  | |  |
