# Supplementary material for: Piecewise Disassembly of a Large-Herbivore Community across a Rainfall Gradient: The UHURU Experiment
Source: PLoS One. 2013 Feb 6;8(2):e55192. doi: 10.1371/journal.pone.0055192 (PMC3566220; doi:10.1371/journal.pone.0055192)
Supplement: Text S1 — Detailed descriptions of soil profiles at each site. (DOCX) [file pone.0055192.s010.docx]

**Text S1**

**Details of soil profiles**

**Piecewise Disassembly of a Large-Herbivore Community across a Rainfall Gradient: The UHURU Experiment**

Jacob R. Goheen, Todd M. Palmer, Grace K. Charles, Kristofer M. Helgen, Stephen N. Kinyua, Janet E. Maclean, Benjamin L. Turner, Hillary S. Young, Robert M. Pringle

**Profile Description: UHURU North (low-rainfall site)**

*Classification*: ALFISOL: Clayey-skeletal, isothermic, semiactive, Typic Haplustalfs

*Profile location*: UHURU North exclosures, 20 m south of the most southerly exclosure and 30 m west of the road

*Date and season*: 22 October 2010; beginning of the wet season

*Elevation:* ~1800 m above sea level

*Slope and site position*: Relatively flat area, sloping slightly towards the north

*Soil moisture regime*: Ustic (~440 mm y^-1^)

*Soil temperature regime*: Isothermic

*Parent material*: Precambrian gneiss

*Vegetation*: Bare ground with sparse grass and scattered acacias (*A. mellifera*)

*Drainage*: Well-drained, although compaction may promote surface runoff

*Surface features*: Erosion of topsoil leaving small quartz fragments on surface

*Faunal activity*: None observed in soil

*Rooting depth*: Abundant in subsoil, but limited below 61 cm

*Coarse fragments*: Common weathered gneiss fragments in the argillic horizon

*Notes:* Color values are for moist soil unless stated and textures are field textures (see analytical information for particle size analysis)

*Diagnostic horizons and features*: (i) Ustic moisture regime

(ii) Ochric epipedon from 0 to 22 cm

(iii) Argillic horizon from 22 to 134 cm

(iv) Clayey-skeletal particle-size control section

(v) Paralithic contact at 134 cm

**General features of the soil:** The soil is an Alfisol because it has an argillic horizon with a base saturation > 35%. It is an Ustalf because of the ustic moisture regime. In the absence of other diagnostic features (notably a kandic horizon) the soil classifies as Typic Haplustalfs. Note that in the absence of information on moisture status at depth in this profile, we do not consider the aridic/udic designations. Similarly, we do not give a mineralogy class in the absence of information on mineralogy. There is little evidence for clay movement (e.g., clay films), but clay depletion in the upper horizons and clear enrichment at depth supports the designation of the subsoil as argillic. The argillic horizon is extremely gravelly, with >90% gneiss fragments. The soil appears degraded through overgrazing, with compacted surface horizons showing platy structure and extreme excavation resistance. This may impede root growth, although there are many fine and very fine roots in the upper part of the argillic horizon. This is presumably because exchangeable base cation concentrations are greater in subsoil, which indicates that analysis of surface soil may not adequately capture the nutrient status of the site. The surface soil is slightly acid with low concentrations of organic matter, exchangeable bases, and a low effective cation exchange capacity. Aluminum saturation is very low (< 1%). The soil contains moderate concentrations of total phosphorus.

**Horizon description:**

A—0 to 4 cm; dark reddish brown (5YR 3/3; 7.5YR 4/4 dry) loamy sand; fine platy structure at the surface and difficult to penetrate, but friable once excavated and breaking to fine granular; common very fine and few fine roots; no clay films; few small (5–20 mm) quartz stones on surface; smooth abrupt boundary.

AB—4 to 13 cm; very dark brown (7.5YR 2.5/3; 7.5YR 3/4 dry) fine sandy clay; extremely hard penetration resistance (requiring a knife or geology hammer to excavate); extremely hard medium platy structure, breaking to medium and fine subangular blocky and fine granular; sticky and very plastic; few fine and very fine roots; no clay films or coarse fragments; many fine (< 1 mm) orange quartz grains; smooth clear boundary.

BA—13 to 22 cm; very dark brown (7.5YR 2.5/3; 5YR 3/4 dry) sandy clay loam; very coarse subangular blocky structure; extremely hard excavation resistance, although crushable in hands once excavated and breaking quickly when wetted; dry; slightly sticky and slightly plastic; common very fine and few fine and medium roots; no clay films; few small (5–20 mm) subrounded quartz stones; smooth clear boundary.

Bt1—22 to 61 cm; extremely gravelly dark reddish brown (2.5YR 3/3; 5YR 3/4 dry) sandy clay loam; > 90% coarse fragments, consisting of ~30% stones (> 7.5 cm diameter), ~30% coarse gravel (2.0–7.5 cm diameter), and ~35% fine and medium gravel (0.2–2.0 cm diameter); mainly subangular quartz stones; many very fine and common fine roots; slightly sticky; common black manganese films on coarse fragments; gradual wavy boundary.

Bt2—61 to 134 cm; extremely gravelly dark red (2.5YR 3/6) clay; > 90% fine and medium gravel (0.2–2 cm diameter) with dark red (2.5YR 3/6) exteriors and strong brown (7.5YR 5/8) interiors; many small hard black angular manganese concentrations; very few fine and very fine roots.

R—partially-weathered gneiss.

**Laboratory analysis: UHURU North (low-rainfall site)**

| Horizon | Designation | Depth | BD | Sand | Silt | Clay | Textural class |
| --- | --- | --- | --- | --- | --- | --- | --- |
|  |  | —cm— | —g cm^-3^— | —%— | —%— | —%— |  |
| 1 | A | 0–4 | 1.55 | 83.4 | 7.7 | 8.9 | Loamy sand |
| 2 | AB | 4–13 | 1.58 | 69.0 | 20.4 | 10.6 | Sandy loam |
| 3 | BA | 13–22 | – | 59.1 | 30.7 | 10.3 | Sandy loam |
| 4 | Bt1 | 22–61 | – | 37.1 | 13.4 | 49.5 | Clay |
| 5 | Bt2 | 61–134 | – | 40.5 | 10.9 | 48.6 | Clay |

| Horizon | ———————Soil pH——————— | | | Total C | Total N | C:N | Total P |
| --- | --- | --- | --- | --- | --- | --- | --- |
|  | Water | CaCl_2_ | BaCl_2_ | —%— | —%— |  | mg P kg^-1^ |
| 1 | 6.4 | 5.5 | 5.3 | 0.91 | 0.05 | 18.2 | 350.2 |
| 2 | 6.0 | 5.2 | 5.1 | 1.53 | 0.07 | 20.5 | 361.9 |
| 3 | 6.2 | 5.7 | 5.2 | 1.35 | 0.07 | 20.4 | 395.1 |
| 4 | 6.5 | 6.0 | 5.8 | 1.07 | 0.07 | 14.4 | 490.9 |
| 5 | 6.0 | 5.3 | 5.2 | 0.46 | 0.02 | 18.6 | 371.4 |

| Horizon | Al | Ca | Fe | K | Mg | Mn | Na | TEB^1^ | ECEC^2^ | BS^3^ |
| --- | --- | --- | --- | --- | --- | --- | --- | --- | --- | --- |
|  | ———————————————————cmol_c_ kg^-1^—————————————————— | | | | | | | | | —%— |
| 1 | 0.02 | 2.51 | 0.01 | 0.78 | 1.29 | 0.07 | 0.01 | 4.6 | 4.7 | 97.8 |
| 2 | 0.02 | 4.61 | 0.01 | 0.96 | 2.35 | 0.06 | 0.02 | 7.9 | 8.0 | 98.9 |
| 3 | 0.02 | 6.46 | 0.01 | 1.03 | 3.11 | 0.03 | 0.03 | 10.6 | 10.7 | 99.5 |
| 4 | 0.04 | 10.31 | 0.01 | 0.69 | 5.11 | 0.04 | 0.05 | 16.2 | 16.2 | 99.5 |
| 5 | 0.04 | 5.70 | 0.01 | 0.14 | 5.12 | 0.04 | 0.15 | 11.1 | 11.2 | 99.2 |

^1^ Total exchangeable bases (i.e. sum of Ca, K, Mg, Na).

^2^ Effective cation exchange capacity (i.e. sum of cations extracted in 0.1 M BaCl_2_).

^3^ Base saturation (i.e. TEB / ECEC × 100).

**Profile description: UHURU Central (intermediate-rainfall site)**

*Classification*: ALFISOL: Clayey-skeletal, isothermic, semiactive, Typic Haplustalfs

*Profile location*: UHURU Central exclosures, between two blocks, 30 m west of the road

*Date and season*: 21 October 2010; beginning of the wet season

*Elevation:* ~1800 m a.s.l.

*Slope and site position*: Relatively flat, sloping very slightly towards the east

*Soil moisture regime*: Ustic (~580 mm y^-1^)

*Soil temperature regime*: Isothermic

*Parent material*: Precambrian gneiss

*Vegetation*: Bare ground with sparse grass and scattered acacias (*A. mellifera* and *A. etbaica*)

*Drainage*: Well-drained, although surface capping may promote surface runoff

*Surface features*: Erosion of topsoil leaving small quartz fragments on surface

*Faunal activity*: None observed in soil

*Rooting depth*: Fine and very fine roots throughout the profile to bedrock

*Coarse fragments*: Common subangular and angular gravel in the subsoil

*Notes:* Color values are for moist soil unless stated and textures are field textures (see analytical information for particle size analysis)

*Diagnostic features*: (i) Ustic moisture regime

(ii) Ochric epipedon from 0 to 4 cm

(iii) Argillic horizon from 4 to 46 cm

(iv) Clayey-skeletal particle control section

(v) Lithic contact at 99 cm

**General features of the soil:** The soil is an Alfisol because it has an argillic horizon with a base saturation > 35%. It is an Ustalf because of the ustic moisture regime. In the absence of other diagnostic features (notably a kandic horizon) the soil classifies as Typic Haplustalfs. Note that in the absence of information on moisture status at depth in this profile, we do not consider the aridic/udic designations. Similarly, we do not give a mineralogy class in the absence of information on mineralogy. There is little evidence for clay movement (e.g., clay films), but clay depletion in the upper horizons (including skeletans) and clear enrichment at depth supports the designation of the subsoil as argillic. The argillic horizon has strongly contrasting particle-size classes, with a clayey upper part (from 4 to 25 cm) and a clayey-skeletal lower part (from 25 to 46 cm); however, it is not clayey over clayey-skeletal because there is < 25% difference in clay concentration in the fine earth fraction in the two parts of the horizon. The subsoil is extremely gravelly, with > 90% gneiss fragments. Manganese concentrations are present in abundance. The soil appears degraded through overgrazing, with compacted surface horizons showing platy structure and extreme excavation resistance. This may impede root growth, although there are fine and very fine roots throughout the subsoil, although to a lesser extent than the north profile. The surface soil is more acidic than the north and south profiles (moderately acid compared to slightly acid in the north and south profiles), but aluminum saturation remains low (≤ 2%). There are low concentrations of organic matter (< 1%) and exchangeable bases, and a low effective cation exchange capacity. Total phosphorus concentrations are lower than in the other profiles.

**Horizon description:**

A—0 to 4 cm; dark reddish brown (5YR 3/4; 5YR 4/6 dry) sandy clay loam; moderate platy structure (especially at surface) breaking to fine granular; moist, compact, friable to firm; very slightly sticky, not plastic; few fine and very fine roots, especially near small grass rhizomes; skeletans and clean quartz grains; no coarse fragments; smooth abrupt boundary.

Bt1—4 to 15 cm; dark reddish brown (2.5YR 3/4; 5YR 3/3 dry) sandy clay; moderate coarse subangular blocky structure, tending to wedge shaped; moist and friable; slightly sticky and very plastic; few fine and very fine roots; few ~2 mm diameter tubular pores that look like ant tunnels; no clay films; few small rounded hard (~1 cm) quartz stones; smooth clear boundary.

Bt2—15 to 25 cm; dark reddish brown (2.5YR 3/4; 5YR 4/6 dry) gritty clay; moderate coarse subangular blocky structure, breaking to fine granular; moist and friable; slightly sticky and very plastic; common very fine and few fine and medium roots, with very fine roots proliferating around small stones; no clay films; common (0.5–2.0 cm) rounded quartz stones; common small (~0.5 cm diameter) hard black manganese concentrations; smooth clear boundary.

Bt3—25 to 46 cm; extremely gravelly yellowish red (5YR 4/6 dry) sandy clay loam; ~90% medium subangular gravel (0.5–1.0 cm diameter), with red (2.5YR 4/6) and yellowish red (5YR 4/6) surfaces; common prominent medium gravel sized black manganese concentrations; few fine and very fine roots; smooth gradual boundary.

BC—46 to 99 cm; extremely gravelly; ~95% medium angular gravel (~1.0 cm diameter); few manganese concentrations as above; white and brown colors on faces; few fine and very fine roots throughout; few small patches of fine earth ~2 cm diameter; very hard penetration resistance; abrupt boundary.

R—99+ cm; hard gneiss

**Laboratory analysis: UHURU Central (intermediate-rainfall site)**

| Horizon | Designation | Depth | BD | Sand | Silt | Clay | Textural class |
| --- | --- | --- | --- | --- | --- | --- | --- |
|  |  | —cm— | —g cm^-3^— | —%— | —%— | —%— |  |
| 1 | A | 0–4 | 1.60 | 70.3 | 19.6 | 10.2 | Sandy loam |
| 2 | Bt1 | 4–15 | 1.55 | 53.8 | 23.6 | 22.6 | Sandy clay loam |
| 3 | Bt2 | 15–25 | – | 43.2 | 8.2 | 48.6 | Clay |
| 4 | Bt3 | 25–46 | – | 45.5 | 19.0 | 35.4 | Sandy clay loam |
| 5 | BC | 46–99 | – | – | – | – | – |

| Horizon | ———————Soil pH—————— | | | Total C | Total N | C:N | Total P |
| --- | --- | --- | --- | --- | --- | --- | --- |
|  | Water | CaCl_2_ | BaCl_2_ | —%— | —%— |  | mg P kg^-1^ |
| 1 | 5.8 | 5.0 | 5.0 | 0.82 | 0.07 | 11.6 | 204.6 |
| 2 | 5.9 | 4.7 | 4.7 | 0.84 | 0.08 | 11.0 | 223.7 |
| 3 | 5.6 | 4.6 | 4.7 | 0.85 | 0.08 | 10.7 | 214.1 |
| 4 | 5.8 | 4.8 | 4.8 | 0.31 | 0.03 | 9.5 | 349.1 |
| 5 | 6.5 | 5.6 | – | 0.42 | 0.04 | 10.1 | 163.1 |

| Horizon | Al | Ca | Fe | K | Mg | Mn | Na | TEB^1^ | ECEC^2^ | BS^3^ |
| --- | --- | --- | --- | --- | --- | --- | --- | --- | --- | --- |
|  | ———————————————————cmol_c_ kg^-1^——————————————————— | | | | | | | | | —%— |
| 1 | 0.07 | 3.33 | 0.01 | 0.68 | 1.93 | 0.10 | 0.02 | 6.0 | 6.1 | 97.2 |
| 2 | 0.15 | 3.92 | <0.01 | 1.24 | 2.44 | 0.04 | 0.06 | 7.7 | 7.9 | 97.5 |
| 3 | 0.20 | 4.99 | <0.01 | 0.85 | 3.65 | 0.04 | 0.04 | 9.5 | 9.8 | 97.5 |
| 4 | 0.09 | 4.19 | <0.01 | 0.14 | 3.57 | 0.13 | 0.17 | 8.1 | 8.3 | 97.3 |
| 5 | – | – | – | – | – | – | – | – | – | – |

^1^ Total exchangeable bases (i.e. sum of Ca, K, Mg, Na).

^2^ Effective cation exchange capacity (i.e. sum of cations extracted in 0.1 M BaCl_2_).

^3^ Base saturation (i.e. TEB / ECEC × 100).**Profile description: UHURU South (high-rainfall site)**

*Classification*: ALFISOL: Fine, isothermic, active, Typic Haplustalfs

*Profile location*: UHURU South exclosures, ~ 20 m west of the road near block three

*Date and season*: 19 October 2010; beginning of the wet season

*Elevation:* ~1800 m a.s.l.

*Slope and site position*: Relatively flat area, sloping gently towards the west, < 1 km from base of the phonolite scarp to the west

*Soil moisture regime*: Ustic (~640 mm y^-1^)

*Soil temperature regime*: Isothermic

*Parent material*: Precambrian gneiss

*Vegetation*: Wooded savanna; irregular grass cover (~80%) with common acacias

*Drainage*: Well-drained, as indicated by a clear wetting front to 25 cm following overnight rainfall

*Surface features*: Small quartz fragments on surface; few larger boulders of red/white quartz

*Faunal activity*: None observed in soil, although small old arthropod nest cavity in profile

*Rooting depth*: Throughout the profile and into weathered rock at > 83 cm

*Coarse fragments*: Scattered rounded quartz boulders on surface and common weathered gneiss fragments in the subsoil

*Notes:* Color values are for moist soil unless stated and textures are field textures (see analytical information for particle size analysis)

*Diagnostic horizons and features*: (i) Ustic moisture regime

(ii) Ochric epipedon from 0 to 21 cm

(iii) Argillic horizon from 21 to 32 cm

(iv) Paralithic contact at 56 cm, lithic contact at 83 cm

**General features of the soil:** The soil is an Alfisol because it has an argillic horizon with a base saturation > 35% (minimum thickness of an argillic horizon is 7.5 cm). It is an Ustalf because of the ustic moisture regime. In the absence of other diagnostic features (notably a kandic horizon) the soil classifies as Typic Haplustalfs. In particular, it does not meet the criteria for ‘inceptic’, because although the argillic horizon is < 35 cm thick, there is lithic contact < 1 m from the soil surface. Note that in the absence of information on moisture status at depth in this profile, we do not consider the aridic/udic designations. Similarly, we do not give a mineralogy class in the absence of information on mineralogy. There is little evidence for clay movement (e.g., absent or weak clay films), but clay depletion in the upper horizons and clear enrichment in the 21 to 32 cm horizon supports the designation of this horizon as argillic. The soil appears degraded through overgrazing, although the surface horizon is not compacted as in the north and central profiles, and rainfall infiltrates rapidly (as shown by a wetting front at depth following rain). The pH is slightly acid at the surface and aluminum saturation is low (< 1%). However, the pH increases markedly at depth, becoming strongly alkaline (pH 8.7) at > 56 cm. There are low concentrations of organic matter (< 1%), but exchangeable bases and effective cation exchange capacity are high compared to the north and central profiles. The abundance of fine and very fine roots in subsoil, including the weathering front, indicate that plants are obtaining nutrients from depth and therefore that analysis of surface soils may not adequately represent the nutrient status of the site. Manganese films and concentrations are common throughout the profile. This profile has more clay at the surface than other UHURU sites, as well as a profile to the east close to the Mpala village (Augustine exclosure). Together with the alkaline subsoil and the considerably greater concentrations of exchangeable bases and effective cation exchange capacity, this suggests that the area around the south exclosures has received input of material from the nearby escarpment, either during the original emplacement of the phonolite lava flows, or subsequently via runoff or dust deposition. (Soils on the plateau are clay-rich Vertisols, with alkaline subsoils containing high concentrations of carbonates.) This may in turn explain the presence of *Acacia drepanolobium* at the southern UHURU site; this species dominates the tree community on the plateau Vertisols.

**Horizon description:**

A—0 to 6 cm; dark reddish brown (5YR 3/4; 5YR 4/6 dry) sandy clay; moderate to strong coarse subangular blocky structure, breaking to fine and very fine subangular blocky and fine granular; slightly sticky and very plastic; upper surface dry and friable, remainder moist and firm; common very fine roots; common very fine black manganese films; no clay films; coarse quartz grains on surface; abrupt smooth boundary.

AB—6 to 21 cm; dark reddish brown (5YR 3/3 moist and dry) fine sandy clay; moderate medium and fine subangular blocky structure, breaking to fine and very fine granular; moist and friable; sticky and very plastic; common very fine and very few fine roots; no clay films; few very fine black manganese films; clear boundary.

Bt—21 to 32 cm; dark reddish brown (2.5YR 3/3; 5YR 4/4 dry) sandy clay; strong coarse, medium, and fine subangular blocky structure; moist part friable and breaking to fine granular; dry and hard part breaking to fine and very fine subangular blocky; sticky and very plastic; common very fine roots; very weak clay films, with most quartz grains clean; pressure faces on ped surfaces; clear smooth boundary.

Bw1—32 to 44 cm; dark reddish brown (2.5YR 3/4; 5YR 4/4 dry) sandy clay loam; strong medium and fine subangular blocky structure, breaking to very fine subangular blocky and fine granular; dry and slightly hard– hard (peds varied); plastic and slightly sticky; few very fine and very few fine roots flattened between ped faces; patches of many very fine roots and a few thicker *Acacia* roots; no clay films; clear smooth boundary.

Bw2—44 to 56 cm; dark reddish brown (2.5YR 3/4; 5YR 4/4 dry) sandy clay loam; strong coarse and medium subangular blocky structure; dry and very hard; plastic and slightly sticky; common very fine and very few fine roots; no clay films; few small (1 cm diameter) rounded quartz pebbles; small abandoned termite nest (5 cm diameter) and associated 1 cm wide tunnel with black organic-rich lining < 1 mm thick; clear smooth boundary.

Cr—56 to 100 cm; weathered and fractured gneiss bedrock; colors varying from white/light brown to black, with strong manganese concentrations diffused throughout thick (1 cm) concentric layers around less weathered gneiss; weathering in bands; much of the horizon containing many black manganese concentrations; mostly small (1–2 cm), subangular, crushable fragments of weathered rock, with dark reddish (2.5YR 3/4) fine earth between and on outside; larger fragments > 2 cm diameter have ~2–4 mm thick yellow/orange sandy rinds, with black manganese concentrations in the layer underneath; common very fine roots throughout the horizon, with few fine roots; abrupt wavy boundary.

R— 83 to 100+ cm; hard and coherent gneiss bedrock; very fine roots penetrating into fractures.

**Laboratory analysis: UHURU South (high-rainfall site)**

| Horizon | Designation | Depth | BD | Sand | Silt | Clay | Textural class |
| --- | --- | --- | --- | --- | --- | --- | --- |
|  |  | —cm— | —g cm^-3^— | —%— | —%— | —%— |  |
| 1 | A | 0–6 | 1.49 | 57.1 | 5.2 | 37.7 | Sandy clay |
| 2 | AB | 6–21 | 1.34 | 49.1 | 28.6 | 22.3 | Loam |
| 3 | Bw1 | 21–32 | 1.31 | 31.0 | 18.5 | 50.5 | Clay |
| 4 | Bw2 | 32–44 | 1.14 | 33.7 | 31.1 | 35.2 | Clay loam |
| 5 | Bw3 | 44–56 | — | 44.1 | 19.5 | 36.4 | Clay loam |
| 6 | Cr | 56–100 | — | 65.9 | 11.5 | 22.6 | Sandy clay loam |

| Horizon | ———————Soil pH——————— | | | Total C | Total N | C:N | Total P |
| --- | --- | --- | --- | --- | --- | --- | --- |
|  | Water | CaCl_2_ | BaCl_2_ | —%— | —%— |  | mg P kg^-1^ |
| 1 | 6.3 | 5.3 | 5.2 | 0.64 | 0.07 | 9.8 | 297.1 |
| 2 | 7.1 | 6.8 | 6.6 | 0.74 | 0.07 | 10.0 | 278.2 |
| 3 | 6.9 | 6.9 | 6.7 | 0.61 | 0.07 | 8.5 | 305.1 |
| 4 | 7.5 | 7.2 | 7.3 | 0.89 | 0.09 | 9.6 | 372.7 |
| 5 | 8.0 | 7.5 | 7.6 | 0.86 | 0.08 | 10.3 | 452.3 |
| 6 | 8.7 | 7.8 | 7.9 | 0.73 | 0.04 | 19.3 | 355.3 |

| Horizon | Al | Ca | Fe | K | Mg | Mn | Na | TEB^1^ | ECEC^2^ | BS^3^ |
| --- | --- | --- | --- | --- | --- | --- | --- | --- | --- | --- |
|  | ——————————————————cmol_c_ kg^-1^——————————————————— | | | | | | | | | —%— |
| 1 | 0.06 | 7.01 | 0.02 | 1.88 | 2.22 | 0.06 | 0.05 | 11.2 | 11.3 | 98.8 |
| 2 | 0.06 | 11.68 | 0.01 | 2.64 | 2.85 | < 0.01 | 0.10 | 17.3 | 17.3 | 99.6 |
| 3 | 0.04 | 18.52 | < 0.01 | 3.45 | 4.31 | 0.01 | 0.21 | 26.5 | 26.5 | 99.8 |
| 4 | 0.05 | 20.87 | 0.01 | 3.69 | 4.43 | < 0.01 | 0.22 | 29.2 | 29.3 | 99.8 |
| 5 | 0.04 | 18.29 | 0.01 | 3.28 | 3.45 | < 0.01 | 0.21 | 25.2 | 25.3 | 99.8 |
| 6 | 0.05 | 11.23 | 0.01 | 3.54 | 2.36 | < 0.01 | 0.23 | 17.4 | 17.4 | 99.6 |

^1^ Total exchangeable bases (i.e. sum of Ca, K, Mg, Na).

^2^ Effective cation exchange capacity (i.e. sum of cations extracted in 0.1 M BaCl_2_).

^3^ Base saturation (i.e. TEB / ECEC × 100).
